# DNA damage repair-related gene signature for identifying the immune status and predicting the prognosis of hepatocellular carcinoma

**DOI:** 10.1038/s41598-023-45999-z

**Published:** 2023-11-03

**Authors:** Yongpan Lu, Sen Wang, Tingting Chi, Yuli Zhao, Huimin Guo, Haizheng Wang, Li Feng

**Affiliations:** 1 Department of Plastic Surgery, Shandong University of Traditional Chinese Medicine, The First Affiliated Hospital of Shandong First Medical University and Shandong Provincial Qian Foshan Hospital, Jingshi Road, Jinan, 250014 Shandong China; 2 Department of Medical Ultrasound, Shandong Medicine and Health Key Laboratory of Abdominal Medical Imaging, The First Affiliated Hospital of Shandong First Medical University and Shandong Provincial Qian Foshan Hospital, Shandong First Medical University, No. 16766, Jingshi Road, Jinan, 250014 Shandong China; 3 Department of Acupuncture and Rehabilitation, The Affiliated Qingdao Hai Ci Hospital of Qingdao University (West Hospital Area), Qingdao, 266000 Shandong China; 4 Department of Medical Ultrasound, Shandong Medicine and Health Key Laboratory of Abdominal Medical Imaging, The First Affiliated Hospital of Shandong First Medical University and Shandong Provincial Qian Foshan Hospital, Jining Medical College, No. 16766, Jingshi Road, Jinan, 250014 Shandong China

**Keywords:** Cancer, Diseases, Oncology

## Abstract

The heterogeneity of hepatocellular carcinoma (HCC) poses a challenge for accurate prognosis prediction. DNA damage repair genes (DDRGs) have an impact on a wide range of malignancies. However, the relevance of these genes in HCC prognosis has received little attention. In this study, we aimed to develop a prognostic signature to identify novel therapy options for HCC. We acquired mRNA expression profiles and clinical data for HCC patients from The Cancer Genome Atlas (TCGA) database. A polygenic prognostic model for HCC was constructed using selection operator Cox analysis and least absolute shrinkage. The model was validated using International Cancer Genome Consortium (ICGC) data. Overall survival (OS) between the high-risk and low-risk groups was compared using Kaplan‒Meier analysis. Independent predictors of OS were identified through both univariate and multivariate Cox analyses. To determine immune cell infiltration scores and activity in immune-related pathways, a single-sample gene set enrichment analysis was performed. The protein and mRNA expression levels of the prognostic genes between HCC and normal liver tissues were also examined by immunohistochemistry (IHC), immunofluorescence (IF) and quantitative real-time PCR (qRT-PCR). A novel ten-gene signature (*CHD1L*, *HDAC1*, *KPNA2*, *MUTYH*, *PPP2R5B*, *NEIL3*, *POLR2L*, *RAD54B*, *RUVBL1* and *SPP1*) was established for HCC prognosis prediction. Patients in the high-risk group had worse OS than those in the low-risk group. Receiver operating characteristic curve analysis confirmed the predictive ability of this prognostic gene signature. Multivariate Cox analysis showed that the risk score was an independent predictor of OS. Functional analysis revealed a strong association with cell cycle and antigen binding pathways, and the risk score was highly correlated with tumor grade, tumor stage, and types of immune infiltrate. High expression levels of the prognostic genes were significantly correlated with increased sensitivity of cancer cells to antitumor drugs. IHC, IF and qRT-PCR all indicated that the prognostic genes were highly expressed in HCC relative to normal liver tissue, consistent with the results of bioinformatics analysis. Ten DDRGs were utilized to create a new signature for identifying the immunological state of HCC and predicting prognosis. In addition, blocking these genes could represent a promising treatment.

## Introduction

Liver cancer is the fourth most frequent cause of cancer-related mortality worldwide and has the sixth highest incidence rate^1^. Most primary liver malignancies are hepatocellular carcinomas (HCCs)^2^. The majority of HCCs are triggered by an underlying liver disease, usually alcoholism or hepatitis B or C virus (HBV or HCV) infection^3^. In China, the 5-year survival rate for patients with HCC, a complicated and diverse illness, is only 14.1% because of the cancer's high incidence of recurrence, which is frequently accompanied by cirrhosis or other comorbidities that make determining the prognosis extremely difficult^4^.

It is well known that the regenerative capacity of the liver is closely linked to the DNA repair process^5–7^. Both mechanisms are substantially dysregulated after chronic liver injury, and the risk of genetic instability is enhanced^8,9^. The DNA damage response (DDR) pathways, which coordinate DNA repair, cell cycle arrest, and eventually cell death or senescence, are affected by this imbalance^10,11^. Hepatic genomic integrity is weakened by abnormalities in DNA repair and related pathways, such as mismatch repair (MMR), homologous recombination (HR), and nonhomologous end joining (NHEJ), which activate hepatocarcinogenesis and result in the development of HCC^12,13^. Given the significance of liver regeneration and the reaction to DNA damage, they could have an additive influence on prognosis and responsiveness to therapy. An increasing number of studies support the use of many proteins to create complete prognosis scores for malignancies based on DNA damage repair. Some DNA damage repair genes (DDRGs) have been employed to forecast the overall survival (OS) of HCC in addition to serum indicators^14^. Recent findings have shown that HCC was categorized into high-repair and low-repair groups based on the expression of DDRGs, and that the high-repair group had a worse prognosis, increased expression of p53 mutation-like genes, and more clinically aggressive features compared to the low-repair group^15^. However, additional research is necessary to determine the exact mechanism by which DDR causes cancer in HCC. Additionally, individualized assessments of the prognosis of the disease can be improved and innovated.

We retrieved the mRNA expression profiles and associated clinical information of HCC patients for this investigation from the TCGA database. Then, we created a prognostic profile of differentially expressed genes (DEGs) linked to DNA damage repair, and we used the ICGC databases to confirm the stability and dependability of the model. A functional enrichment analysis was conducted to explore the underlying processes. Additionally, the relationship between immune infiltration and prognostic gene expression was examined. Furthermore, the association between tumor stemness and cancer chemoresistance based on the expression levels of the prognostic genes was investigated. Finally, experimental validation of the protein and mRNA expression levels of the prognostic genes in HCC and normal tissues was performed.

## Methods

### Data acquisition

To obtain the clinical data and RNA sequencing data for 374 patients with liver cancer, we utilized the TCGA website (https://portal.gdc.cancer.gov/repository). Additionally, samples from an additional 231 HCC patients were collected from the ICGA website (https://dcc.icgc.org/projects/LIRI-JP). We identified 513 DNA damage repair genes (DDRGs) from the Molecular Signatures database (MSigDB, http://www.gsea-msigdb.org/gsea) and prior literature^16^ (Supplementary Table S1).

### Construction and validation of a prognostic DNA damage repair-related gene signature

The “limma” R package was used to identify DEGs between tumor and normal tissues in the TCGA cohort with a fold change > 1 and a false discovery rate < 0.05. Then, we identified DDRGs with prognostic significance using univariate Cox analysis and the Benjamini and Hochberg (BH) correction approach. To reduce the risk of overfitting, LASSO-penalized Cox regression analysis was employed to construct a prognostic model. The LASSO method can shrink the coefficients of some unimportant features to zero, thus improving the selection of features; and it can produce sparse solutions at multicollinearity, which is suitable for multi-dimensional datasets and multicollinear data; moreover, it can simplify the complexity of the model while maintaining a high prediction accuracy^17–19^. The “glmnet” R package with the LASSO algorithm was used to choose and compress variables to obtain regression coefficients precisely equal to zero and an understandable model. The independent variable in the LASSO Cox regression analysis was a matrix of standardized expression levels for potential prognostic DEGs, and the dependent variables were OS and the status of patients in the TCGA cohort. We employed tenfold cross-validation to determine the penalty parameter (λ) for the prognostic model, selecting the value of λ corresponding to the lowest partial likelihood deviation. The DDRG expression levels and their corresponding regression coefficients were used to calculate patient risk scores. The equation was as follows:

$$risk\; score={e}^{sum(each\; gene\text{'}s \; expression \times corresponding\; coefficient)}$$

Using the median risk scores, patients were classified into high-risk and low-risk groups. The “Rtsne” and “ggplot2” R packages were employed to perform PCA and t-SNE analyses to investigate the distribution of the two distinct risk groups. The “survminer” R package was utilized to perform survival analysis. Furthermore, a time-dependent receiver operating characteristic (ROC) curve analysis was conducted to determine the predictive power of prognostic characteristics. Univariate and multivariate Cox analyses were performed to investigate the independent prognostic significance of the 10 genetic markers.

### Functional enrichment analysis

The online websites (http://www.bioinformatics.com.cn/) was utilized to perform Gene Ontology (GO) and Kyoto Encyclopedia of Genes and Genomes (KEGG) analyses of the DEGs in high and low risk groups. Additionally, we used the “gsva” package to perform single-sample gene set enrichment analysis (ssGSEA) to determine the infiltration of diverse immune cells and the activity levels of diverse immune-related pathways in each sample. The BH technique was employed to adjust the P value.

### Analysis of the tumor microenvironment and immune response

We assessed the level of infiltration of immune and stromal cells in distinct tumor tissues using immune and stromal scores^20^. To investigate the relationship between the risk score and immune infiltration subtype, we performed a 2-way ANOVA. To measure the stem cell-like characteristics of tumors, we extracted tumor stem cell features from the transcriptome and epigenetics of TCGA tumor samples^21^.

### Analysis of chemotherapy sensitivity

We accessed the NCI-60 database through the CellMiner interface (https://discover.nci.nih.gov/cellminer), which includes 60 cancer cell lines from 9 different tumor types. Pearson correlation analysis was used to assess the correlation between prognostic gene expression and drug sensitivity. The effectiveness of 217 FDA-approved drugs (Supplementary Table S2) was evaluated using a correlation study.

### Analysis of the human protein atlas (HPA) database

The HPA (http://www.proteinatlas.org/) database allows researchers to access enormous volumes of proteomic and transcriptome data for individual human cells and tissues^22^. Protein expression levels of ten genes were verified by immunohistochemistry (IHC) in the HPA database.

### Patients and tissue samples

All HCC and healthy liver tissue sections were obtained from the Shandong Provincial Hospital of Traditional Chinese Medicine’s Department of Pathology. The Declaration of Helsinki guidelines were followed in the conduct of this investigation. This study was approved by Shandong University of Traditional Chinese Medicine (Approval Number: AF/SC-08/02.0).

### HCC mouse model

BALB/c (4–6 weeks) mice weighing 20 ± 3 g were selected for the construction of the HCC model. After one week of acclimatization feeding, 200 μl of mouse HCC cells (1 × 10^7^ cells/ml resuspended in sterile PBS solution) was injected subcutaneously into the right shoulder of mice. When the tumors had developed to a diameter of approximately 10 mm, the mice were killed with an intraperitoneal injection of 250 mg/kg pentobarbital sodium. Then, the tumor was removed. Controls were normal BALA/c mouse livers without any treatment. Fresh mouse tumor and liver tissues were stored at − 80 °C for quantitative real-time PCR (qRT-PCR). All animal experiments in this study were approved by the Animal Experiments Ethics Committee of The First Affiliated Hospital of Shandong First Medical University, approval umber: SYDWLS(2021)002.

### Immunohistochemistry (IHC) and immunofluorescence (IF) to confirm protein expression differences in prognostic genes between HCC and normal tissues

Tissue slices were deparaffinized, rehydrated, and treated with 0.3% methanol-H_2_O_2_ solution at room temperature for 20 min to quench endogenous peroxidase activity; the slices were then washed three times with PBS. Antigen retrieval was carried out with EDTA treatment for 20 min; the slices were then washed three times with PBS, blocked with 5% goat serum for 30 min, and incubated with primary antibody overnight. On the second day, the slices were warmed, washed three times with PBS, incubated with secondary antibody for 1 h, washed three times with PBS, and developed with DAB for 4 min. Counterstaining was performed with hematoxylin for 1 min, followed by treatment with hydrochloric acid alcohol differentiation solution for 2 s; hematoxylin was “blued” for 10 min. The slices were dehydrated through graded alcohols, immersed in xylene for 10 min, and mounted with neutral resin.

Paraffin sections were dewaxed in water, and the sections were sequentially placed in environmentally friendly dewaxing solution I for 10 min followed by solution II for 10 min. The sections were then washed with anhydrous ethanol I for 5 min, anhydrous ethanol II for 5 min, anhydrous ethanol III for 5 min, and distilled water. Afterward, the antigen was repaired, and it was finished by natural cooling. The slides were submerged in PBS (pH 7.4) and rinsed three times; each time, they were shaken for five minutes on a decolorizing shaker. After being softly shaken dry, the sections were circled around the tissue with a histochemical pen, and 3% BSA was added dropwise for 30 min. After applying the prepared primary antibody dropwise to the sections, they were incubated overnight at 4 °C in a humidified box. The slides were submerged in PBS (pH 7.4) and cleaned three times; each time, they were shaken for five minutes on a decolorizing shaker. On this shaker, the slides were washed three times in PBS (pH 7.4) for five minutes each time. The slides were observed and recorded using a Nikon Eclipse Ti2 confocal microscope (Nikon Instruments (Shanghai) Co., Ltd., Shanghai, China).

The primary antibodies used in our work were as follows: anti-*CHD1L* antibody (DF8521, Affinity), anti-*HDAC1* antibody (AF6433, Affinity), anti-*KPNA2* antibody (DF6510, Affinity), anti-*MUTYH* antibody (DF6500, Affinity), *NEIL3* polyclonal antibody (YT3031, ImmunoWay), anti-*POLR2L* antibody (DF6960, Affinity), anti-*PPP2R5B* rabbit polyclonal antibody (HA500409, HUABIO), *Rad54B* polyclonal antibody (YT3971, ImmunoWay), anti-*RUVBL1* antibody (DF7961, Affinity), and anti-*SPP1* antibody (AF0227, Affinity).

### RNA isolation and qRT-PCR

Total RNA was extracted using RNAiso Plus (9109, Takara, Japan) according to the manufacturer’s protocol. Sketch™ RT Master Mix (RR036A, Takara, Japan) was used to synthesize complementary DNA by reverse transcription of RNA into DNA. Quantitative real-time PCR (qRT-PCR) experiments were performed using TB-Green™ Premix™ II (RR820A, Takara, Japan). Primers were designed and synthesized by Servicebio. GAPDH was used as an endogenous reference gene. Relative gene expression was determined using the 2^−ΔΔCT^ method. The sequences of the employed PCR primers are shown below.

- *GAPDH*-F 5′-CCTCGTCCCGTAGACAAAATG-3′
- *GAPDH*-R 5′-TGAGGTCAATGAAGGGGTCGT-3′
- *HDAC1*-F 5′-CACAAAGCCAATGCTGAGGAG-3′
- *HDAC1*-R 5′-CGATGTCCGTCTGCTGCTTAT-3′
- *KPNA2*-F 5′-AGAACCTTTGATGAACCTCCTGA-3′
- *KPNA2*-R 5′-TTTTATCCAAGCCTCCACACTCT-3′
- *CHD1L*-F 5′-GAAGACCTGAGTTTGGGTGATGT-3′
- *CHD1L*-R 5′-CGCTTGCTTTCTTTTTCTTTGC-3′
- *PPP2R5B*-F 5′-GCGGTATTTGGGACCCTCTA-3′
- *PPP2R5B*-R 5′-TTCTGCTGCTCCTGTTGTTTTT-3′
- *RAD54B*-F 5′-CAGACGAGAATCACCAGCGG-3′
- *RAD54B*-R 5′-CCTAAGCCCATTTCATCAGCAA-3′
- *RUVBL1*-F 5′-AGAGCACCACGAAGACGCA-3′
- *RUVBL1*-R 5′-GCAATAGCAAGGGCCAAGG-3′
- *POLR2L*-F 5′-CAAATGGGAAGCCTACCTGG-3′
- *POLR2L*-R 5′-CAGCTTCTCAATCAGGTCCACG-3′
- *MUTYH*-F 5′-ATCGCCTTTGACCAGGTAACC-3′
- *MUTYH*-R 5′-ATGGCAGCTTGATTGAAGTCCC-3′
- *SPP1*-F 5′-TACAGCCTGCACCCAGATCCTAT-3′
- *SPP1*-R 5′-GCTTTCATTGGAATTGCTTGGA-3′
- *NEIL3*-F 5′-GGCTGCTCCAATGAATGCTAA-3′
- *NEIL3*-R 5′-CTCCCGTGGGTTAATCAAGATG-3′

### Statistical analysis

R (version 4.0.5) and the appropriate packages were employed for statistical analyses. Statistically significant differences were denoted by a P value of < 0.05. To determine differences between two groups, the Wilcoxon test was applied. Kaplan‒Meier curves were used to create survival curves.

### Ethics approval

This study were approved by the ethics committee of Shandong University of Traditional Chinese Medicine (AF/SC-08/02.0) and the Animal Experiments Ethics Committee of The First Affiliated Hospital of Shandong First Medical University, approval umber: SYDWLS(2021)002. All procedures were carried out in strict accordance with the 1964 Declaration of Helsinki guidelines. All patients involved in this study provided informed consent before the study.

## Results

**Figure 1:**
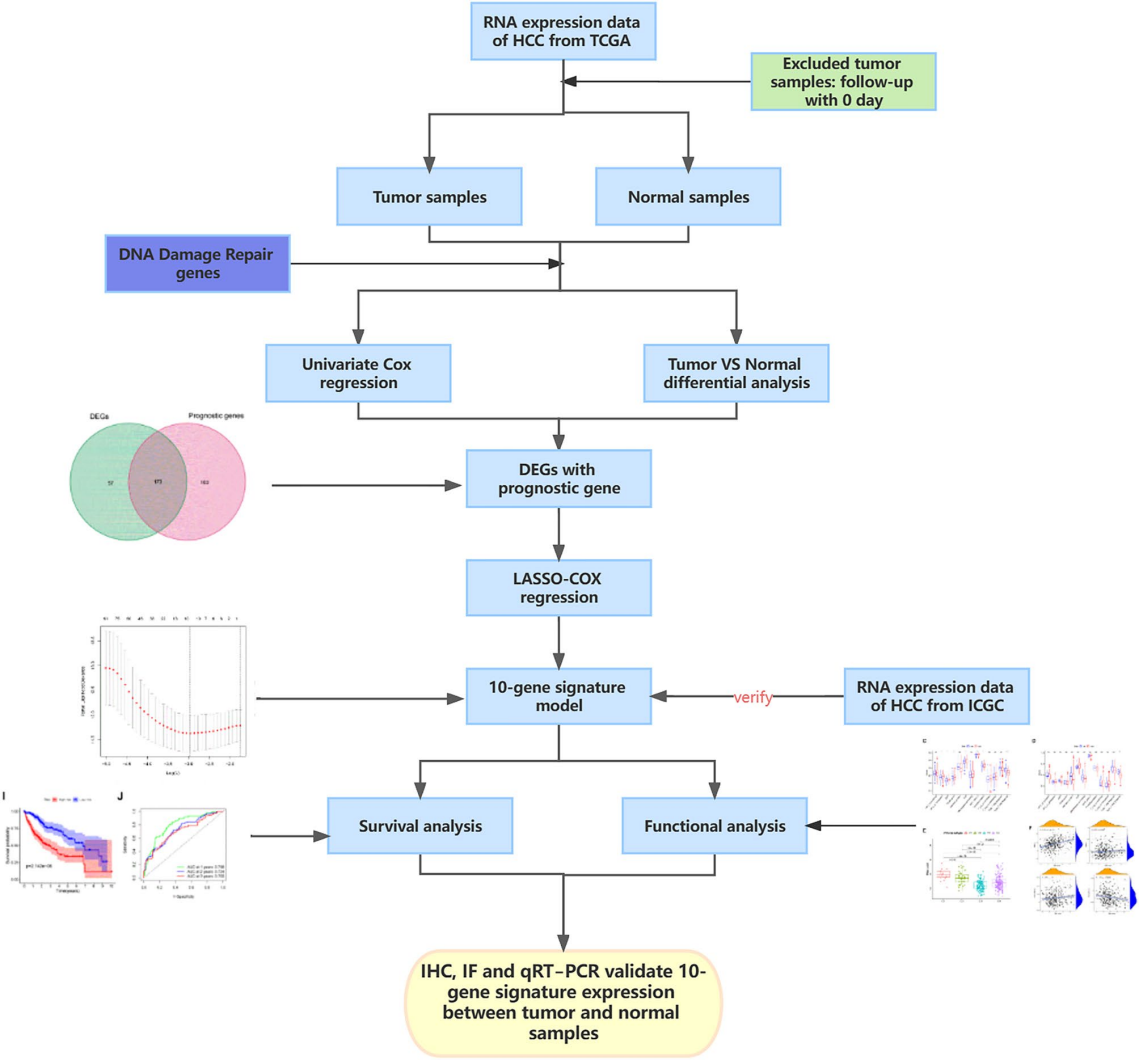
Flow chart of data collection, analysis, and experiments.

This study’s flow chart is shown in Fig. 1. A total of 365 HCC patients from TCGA and 231 HCC patients from ICGC were included in this analysis after several HCC patients with insufficient clinical data were excluded. Supplementary Table S3 provides a summary of the patients' full clinical features.

### Identification of prognostic DNA damage repair-associated DEGs in the TCGA cohort

**Figure 2:**
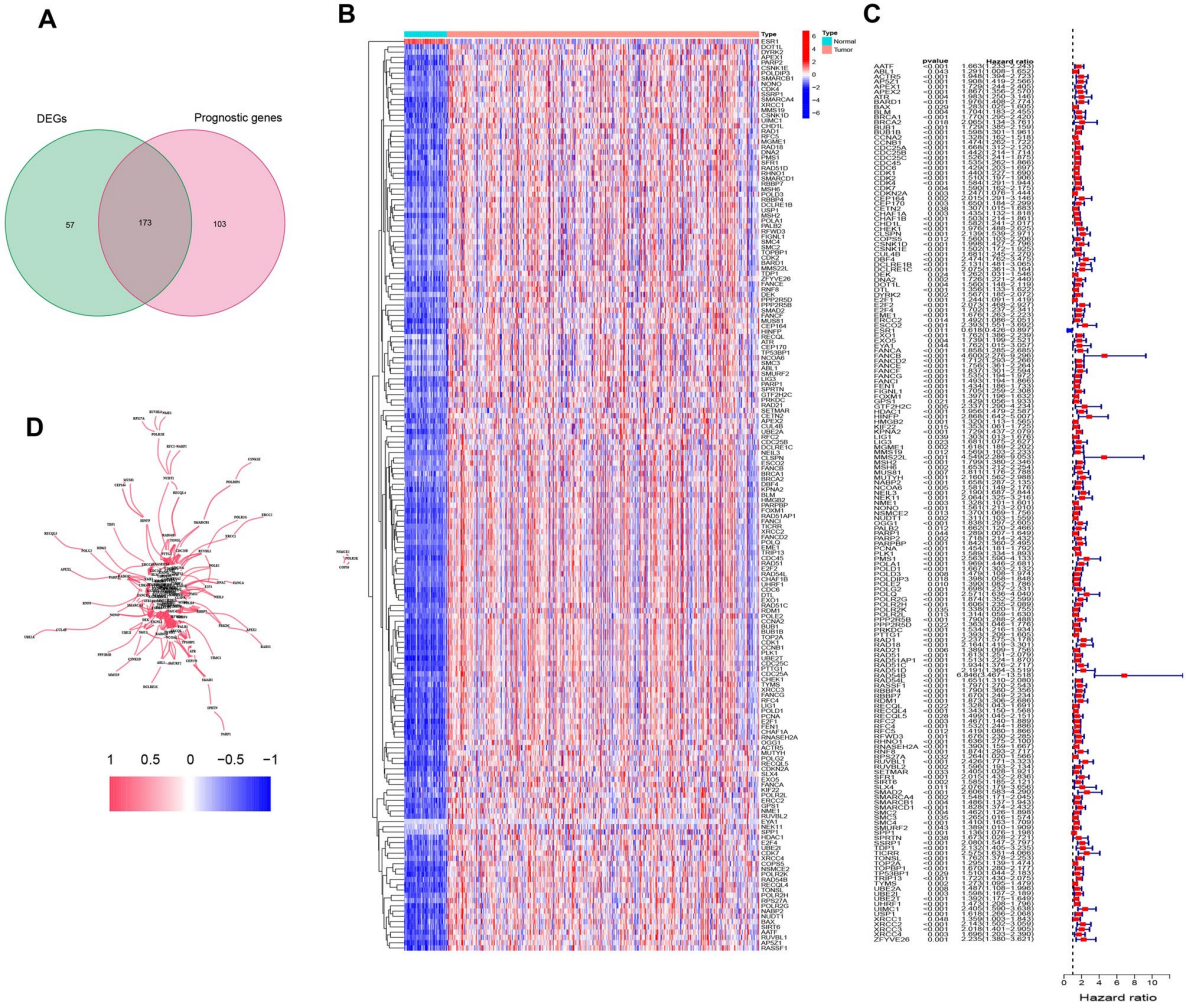
Identification of the candidate DDEGs in the TCGA cohort. (**A**) Venn diagram to identify DEGs between HCC tissues and adjacent normal tissues. (**B**) Expression of 173 overlapping genes between HCC tissues and adjacent normal tissues. (**C**) Forest plots showing the results of the correlation with 173 overlapping genes and OS. (**D**) The correlation network of candidate genes.

A total of 230 DDRGs were expressed differently in HCC tumor tissue compared to normal tissue; 173 of them were associated with OS, according to a univariate Cox analysis (Fig. 2A). The heatmap showed that 173 DDRGs showed differences in normal and HCC tissues, and almost all of them were highly expressed in HCC (Fig. 2B). As prognostic indications, 173 DDRGs were retained, and the risk ratio for the *RAD54B* gene was 6.846 (95% CI 3.467–13.518, P < 0.001, Fig. 2C). Figure 2D displays the association between these genes.

### Building prognostic models in the TCGA cohort

To develop a prognostic model, LASSO-Cox regression analysis was applied to analyze the expression profiles of the 173 genes mentioned earlier. Based on the optimal value of λ, markers for 10 genes were identified (Supplementary Fig. S1). The DDRG signature was calculated as follows:

$$RiskScore=0.051\times {CHD1L}_{expression}+0.018\times {HDAC1}_{expression}+0.151\times {KPNA2}_{expression}+0.131\times {MUTYH}_{expression}+0.165\times {NEIL3}_{expression}+0.050\times {POLR2L}_{expression}+0.146\times {PPP2R5B}_{expression}+0.233\times {RAD54B}_{expression}+0.114\times {RUVBL1}_{expression}+0.032\times {SPP1}_{expression}$$

**Figure 3:**
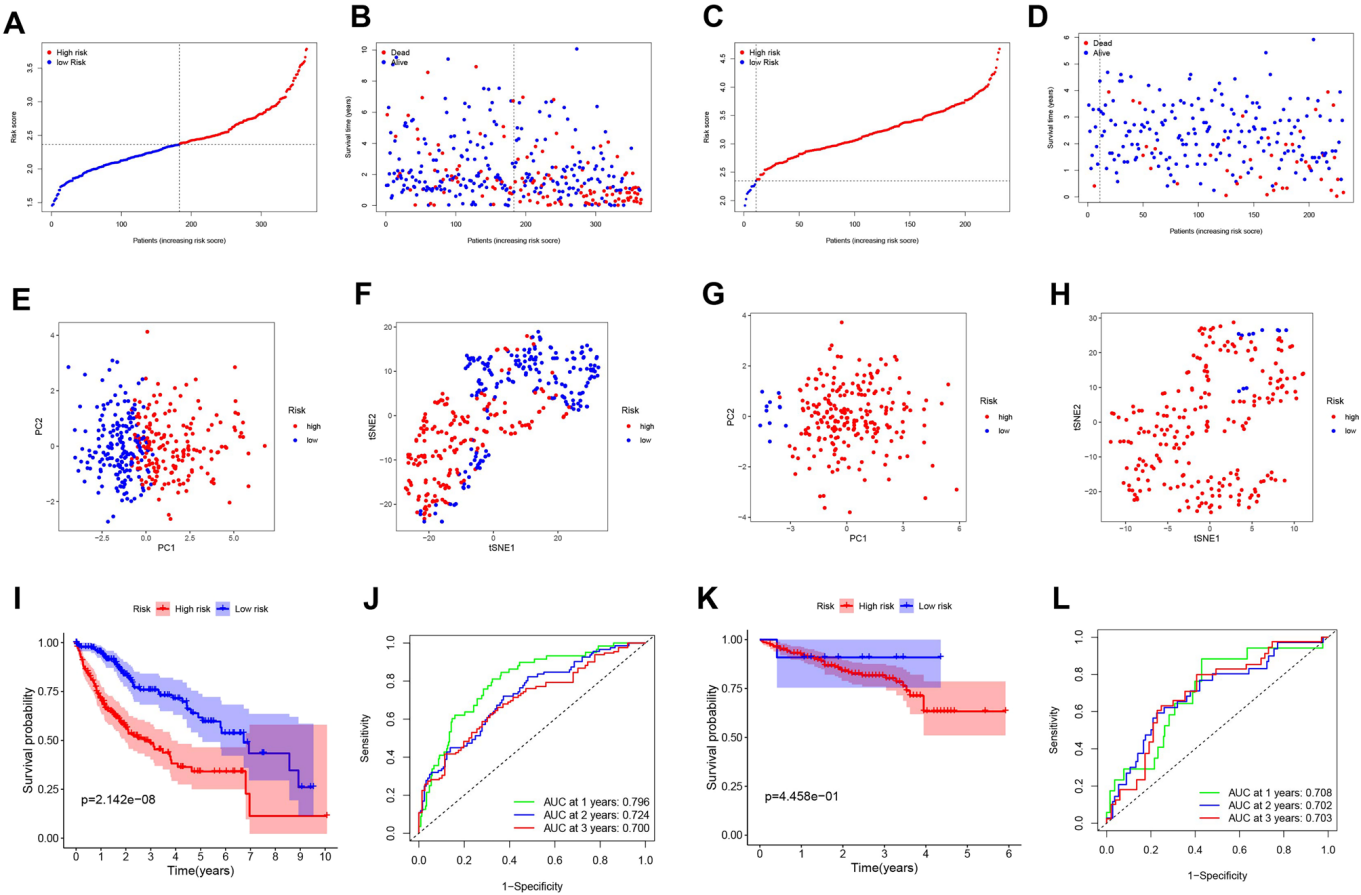
The prognosis analysis of the 10-gene signature model in the TCGA cohort and the ICGC cohort. TCGA cohort (**A**,**B**,**E**,**F**,**I**,**J**), ICGC cohort (**C**,**D**,**G**,**H**,**K**,**L**). (**A**,**C**) The median value and distribution of the risk scores. (**B**,**D**) The distribution of OS status. (**E**,**G**) PCA plot. (**F**,**H**) t-SNE analysis. (**I**,**K**) Kaplan‒Meier curves for OS of patients in the high- and low-risk groups. (**J**,**L**) AUC time-independent ROC curves for OS.

Based on median cutoff values, patients were divided into two groups (Fig. 3A). PCA and t-SNE analyses revealed that patients with high and low risk status were concentrated in two areas (Fig. 3E,F). Furthermore, when risk scores increased, HCC patients were more likely to die prematurely, and survival analysis further revealed that high-risk individuals had shorter OS (Fig. 3B,I). The accuracy of the model for predicting the prognosis of HCC was evaluated using time-dependent ROC curves, which yielded AUCs of 0.796, 0.724, and 0.700 for 1-, 2-, and 3-year predictions, respectively (Fig. 3J). To examine the correlation between the ten prognostic genes and prognosis, a prognostic survival study was conducted, which indicated a significant association between high expression of each gene and poor OS (Supplementary Fig. S2, all P < 0.05). Additionally, the expression levels of these genes were significantly higher in tumor tissues than in normal tissues (all P < 0.001) as demonstrated in Supplementary Fig. S3.

### Validation of the 10-gene signature in the ICGC cohort

To test the stability of the model constructed from the TCGA cohort, patients in the ICGC cohort were also categorized into high- and low-risk groups based on the median value of the TCGA cohort, and our model also had strong predictive value. Fewer patients with low-risk than high-risk HCC in the ICGC cohort (Fig. 3C). The high-risk group had a lower survival duration and early mortality in the ICGC cohort (Fig. 3D,K). PCA and t-SNE analyses were conducted to categorize patients into different risk groups, which were divided into two orientations (Fig. 3G,H). In the ICGC queue, this model still had strong predictive ability (Fig. 3L).

### Independent prognostic value of the 10-gene signature

**Figure 4:**
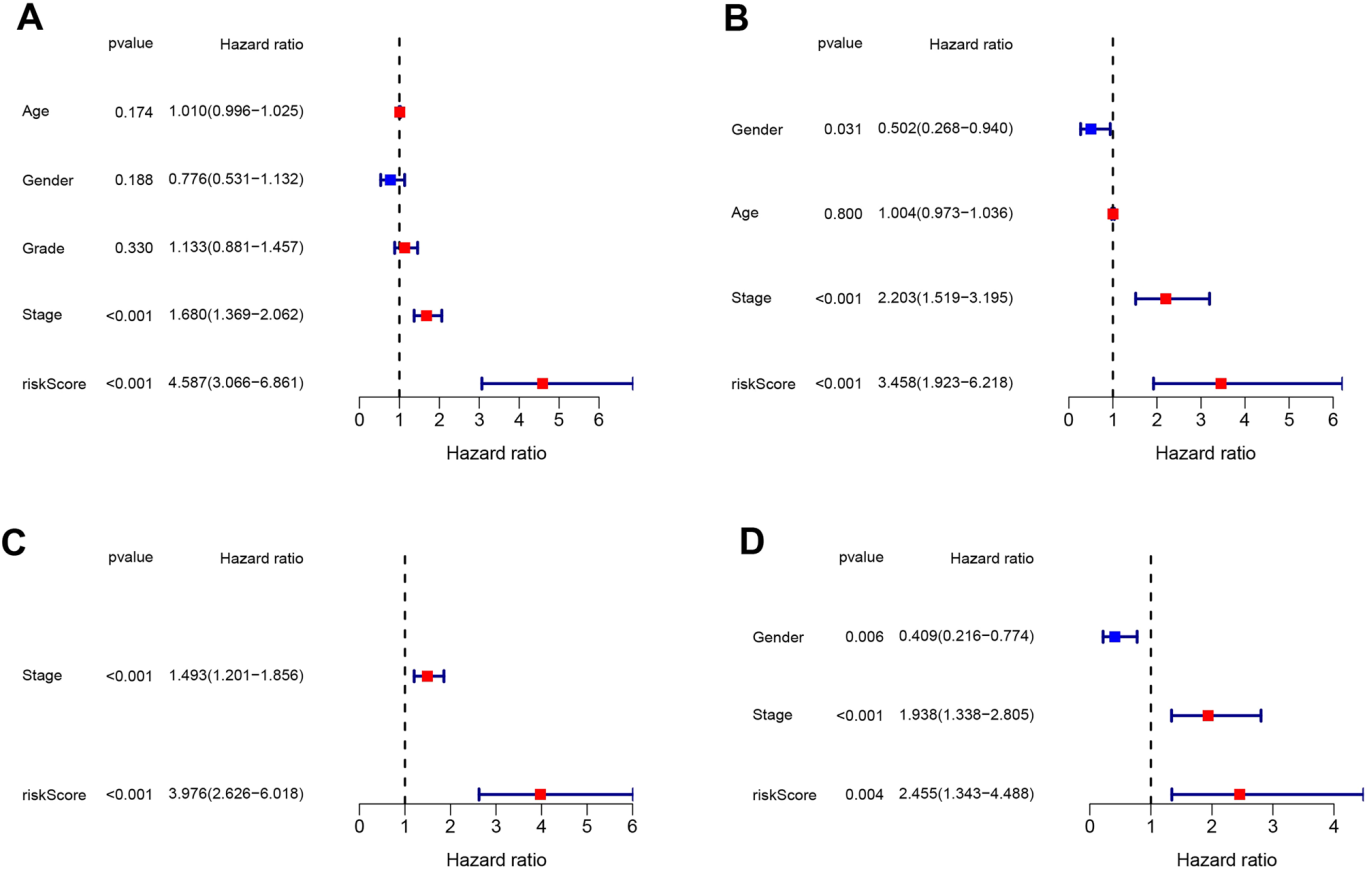
Independence validation of the risk score in the TCGA and ICGA cohorts. (**A**,**B**) TCGA cohorts, (**C**,**D**) ICGC cohorts.

Using univariate and multivariate Cox analyses, we identified the risk score as an independent prognostic factor for OS. Univariate Cox analysis revealed a significant relationship between risk scores and OS (TCGA cohort: HR = 4.587, 95% CI 3.066–6.861, P < 0.001; ICGC cohort: HR: 3.458, 95% CI 1.923–6.218, P < 0.001) (Fig. 4A,B). Multivariate Cox analysis showed that the risk score remained an independent predictor of OS after controlling for other confounders (TCGA cohort: HR = 3.976, 95% CI 2.626–6.018, P < 0.001; ICGC cohort: HR: 2.455, 95% CI 1.343–4.488, P = 0.004) (Fig. 4C,D). Furthermore, we discovered that the stage of HCC was an independent predictor of OS (TCGA cohort: HR = 1.493, 95% CI 1.201–1.856, P < 0.001; ICGC cohort: HR: 1.938, 95% CI 1.338–2.805, P < 0.001). The risk score and clinicopathological characteristics therefore provided strong predictive value for HCC.

### Risk scores and clinical characteristics of prognostic models

We found significantly higher risk scores for grades 3–4 compared to grades 1–2 in HCC (Supplementary Fig. S4D). In both the TCGA database and the ICGC database, risk scores were significantly higher for tumors in stages III–IV compared to tumors in stages I–II (as data about the grade of HCC were not available for the ICGC dataset) (Supplementary Fig. S4C,G). Furthermore, we obtained the relationship between the expression of prognostic genes and HCC staging and grading through the online website (http://vip.sangerbox.com/home.html). The results revealed that the expression of prognostic genes was significantly higher in tumor grades 3–4 than in tumor grades 1–2, except for *POLR2L* and *PPP2R5B* (P < 0.05, Supplementary Fig. S5). In terms of tumor stage, stage III or stage II had higher gene expression than stage I. Except for the genes *CHD1L* and *POLR2L*, there was no difference in the genes in each stage (stage IV was not compared because of the small sample size) (P < 0.05, Supplementary Fig. S6).

### Analysis of immune status and tumor microenvironment

**Figure 5:**
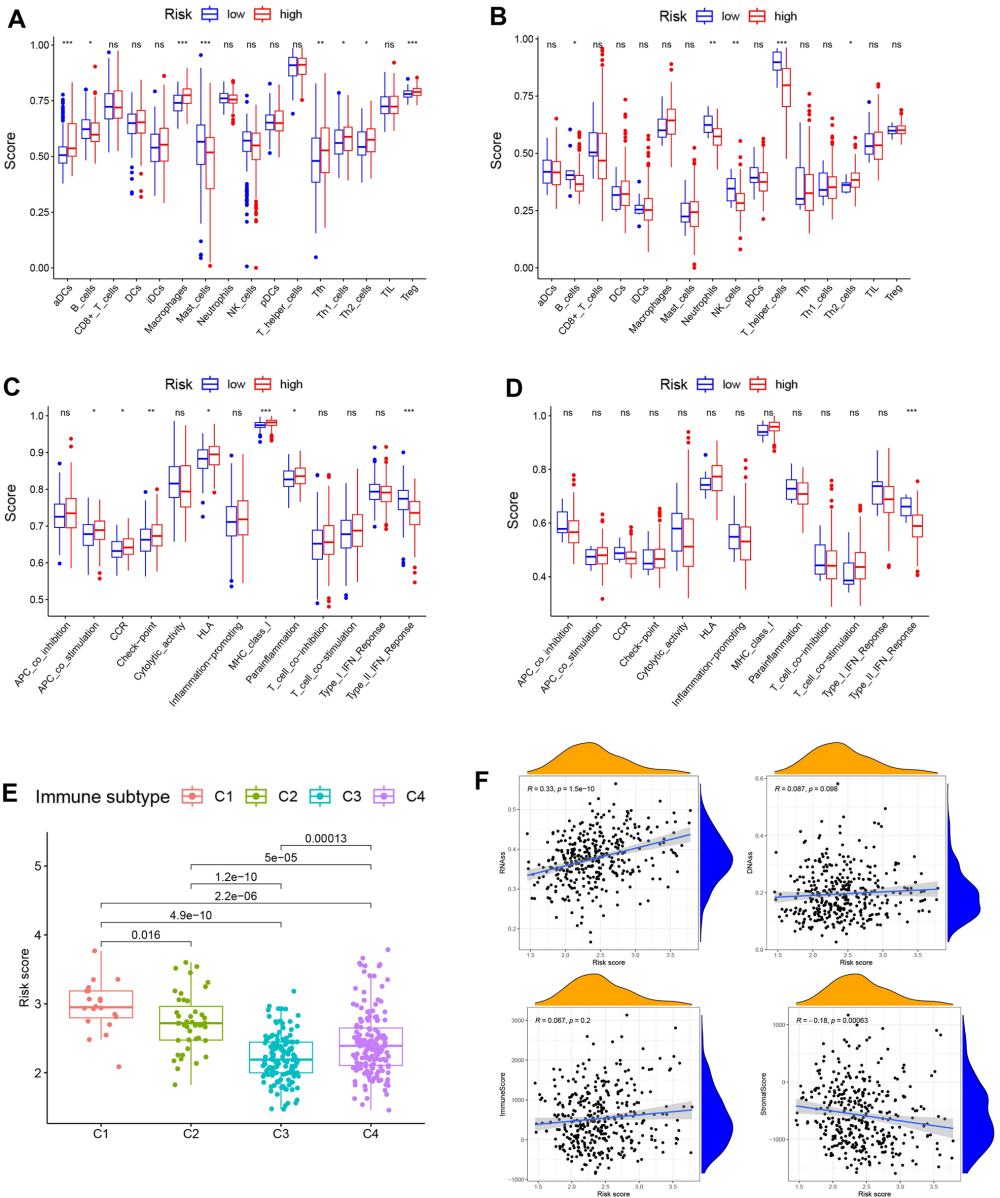
Relationship between immune status and risk scores and the tumor microenvironment between different risk groups. (**A**,**C**) TCGA cohort, (**B**,**D**) ICGC cohort. (**A**,**B**) The scores of 16 immune cells and (**C**,**D**) 13 immune-related functions are shown in boxplots. (**E**) Comparison of the risk score in different immune infiltration subtypes. (**F**) The association between risk score and RNAss, DNAss, stromal score and immune score. P values are shown as follows: *ns* not significant; *P < 0.05; **P < 0.01, ***P < 0.001.

We comprehensively analyzed the relationship between risk scores and immune status. In the TCGA cohort, the high-risk group had significantly higher levels of components associated with the antigen presentation pathway, including aDCs, APC costimulation, HLA, and MHC class I (all adjusted P values < 0.05, as shown in Fig. 5A,C). This finding suggests a potential association between increased risk scores and altered immune activity, specifically within the antigen presentation pathway. Compared to those in the low-risk group, individuals in the high-risk group exhibited increased fractions of Tfh cells, Treg cells, Th 1 cells and Th 2 cells indicating alterations in T-cell regulation between the two groups. Furthermore, the high-risk group exhibited higher CCR, and checkpoint, macrophage activity scores, but the reverse activity was found for the type II IFN response score (adjusted P < 0.05). The outcomes of the comparisons between the two risk categories in the ICGC cohort were comparable to those in the TCGA cohort (adjusted P < 0.05, Fig. 5B,D).

We next examined the correlation between risk scores and immune infiltration. Six immune infiltration subtypes associated with human tumor promotion and tumor suppression were identified: C1 (wound healing), C2 (INF-g dominant), C3 (inflammatory), C4 (lymphocyte depleted), C5 (immunologically quiet) and C6 (TGF-b dominant)^23,24^. Two immune categories—C5 and C6—were excluded from this study due to the limited number of HCC samples available for these subtypes. Our investigation revealed a significant correlation between risk scores and immune infiltration of HCC in the TCGA database, with high-risk scores being strongly associated with the C1 subtype and low-risk scores demonstrating a significant link with the C3 subtype (Fig. 5E).

Neoplastic stemness may be assessed using DNA stemness scores (DNAss) and RNA stemness scores (RNAss) based on DNA methylation patterns and mRNA expression, respectively^25^. Correlation analysis was then conducted to examine whether risk scores were associated with tumor stem cells and the immune microenvironment. The findings revealed that risk scores were not significantly correlated with DNAss or immune scores but instead showed a positive and significant correlation with RNAss and a negative correlation with stromal scores (P < 0.001, Fig. 5F).

### Analyses of immune-related genes

**Figure 6:**
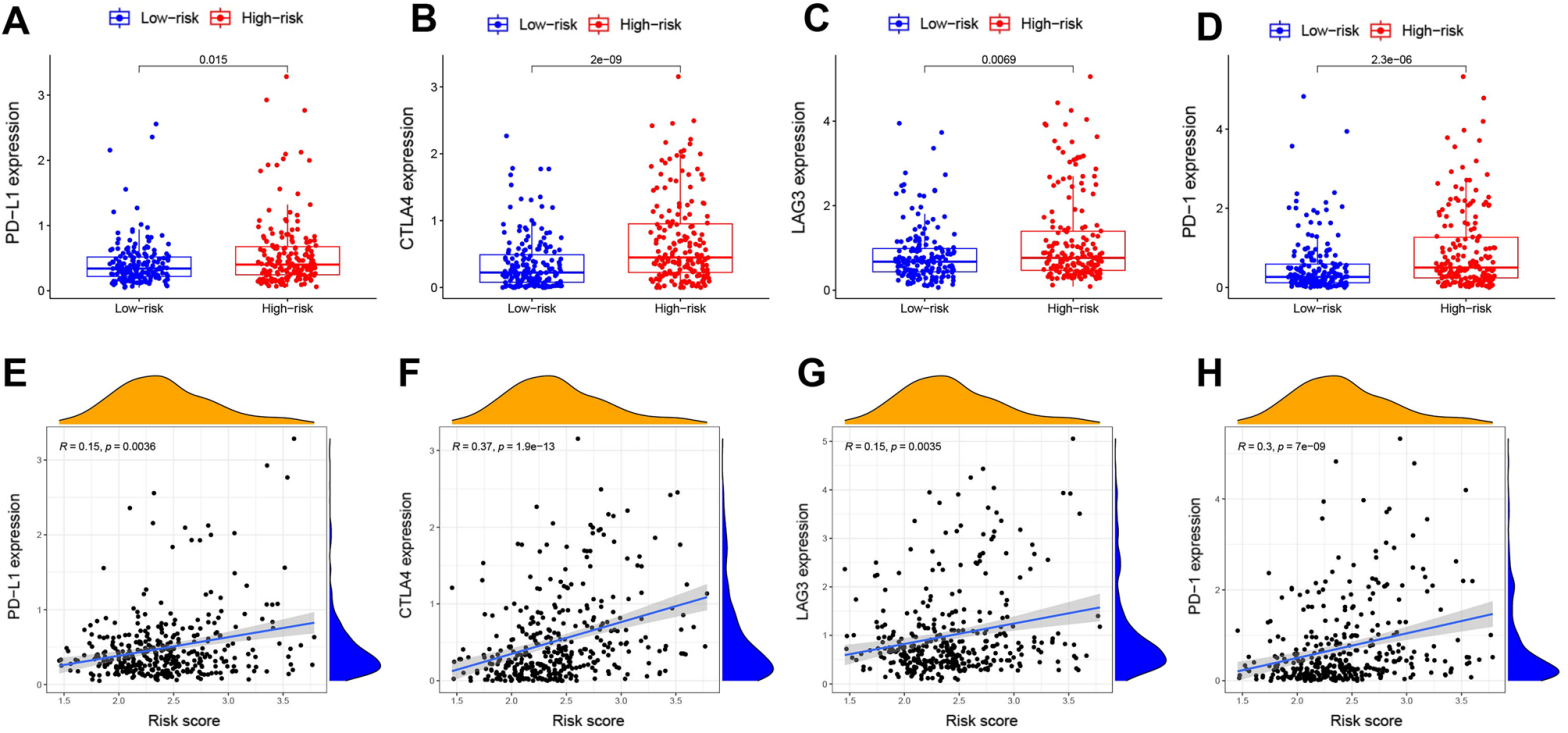
Comparison of the expression levels of PD-L1, CTLA4, LAG3 and PD-1 between different risk groups and correlation analysis between the risk score and the expression levels of PD-L1, CTLA4, LAG3 and PD-1. (**A**,**E**) PD-L1. (**B**,**F**) CTLA4. (**C**,**G**) LAG3. (**D**,**H**) PD-1.

An important regulator of cancer immune evasion is the PD-1/PD-L1 pathway. The expression levels of immunological checkpoints such as PD-1 and PD-L1 are crucial markers for personalized immunotherapy. As expected, the expression levels of PD-1, PD-L1, CTLA4 and LAG3 were found to be significantly higher in the high-risk group than in the low-risk group, with statistically significant differences observed (Fig. 6A–D). Furthermore, our analysis showed that the expression levels of these immune checkpoints were positively correlated with the risk score (Fig. 6E–H).

### Analyses of biological function and pathway enrichment

**Figure 7:**
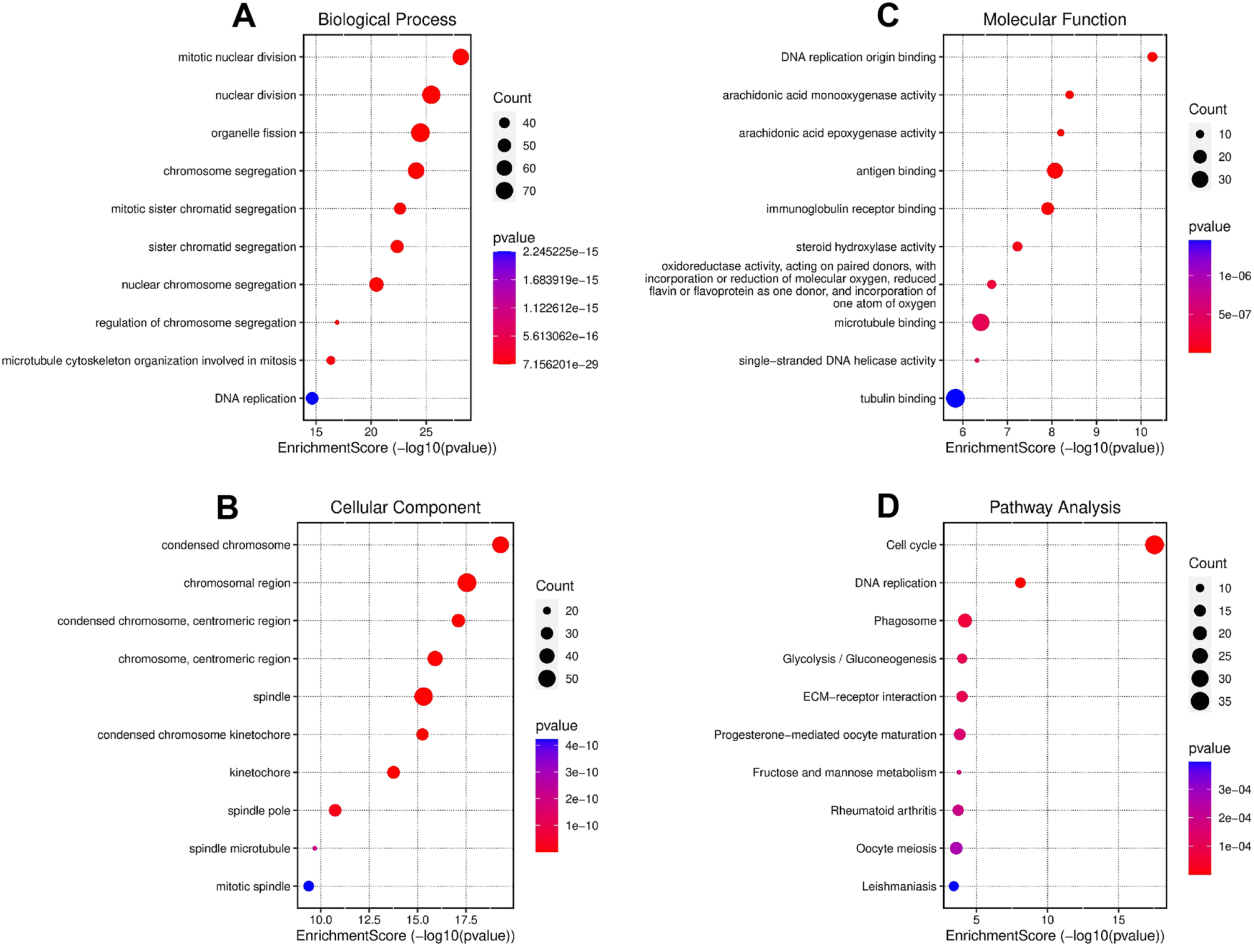
Analysis of Gene Ontology (**A**–**C**) and KEGG pathway enrichment (**D**) in the DEGs in the TCGA database.

KEGG functional enrichment studies were performed utilizing DEGs in high and low risk groups to clarify the underlying pathways that were related to the risk score. DEGs were shown to be abundant in the cell cycle, phagosome, DNA replication, human T-cell leukemia virus 1 infection, and HIF-1 signaling pathways (Fig. 7D, Supplementary Table S4). GO functional enrichment studies were also carried out. The results demonstrated that the DEGs virtually mapped to immune-related GO keywords such as antigen binding, organelle fission, chromosomal region, and tubulin binding (Fig. 7A–C, Supplementary Table S5), indicating that changes in survival across subgroups may be connected to the immunological state of patients.

### Prognostic gene expression and cancer cell sensitivity to chemotherapy

**Figure 8:**
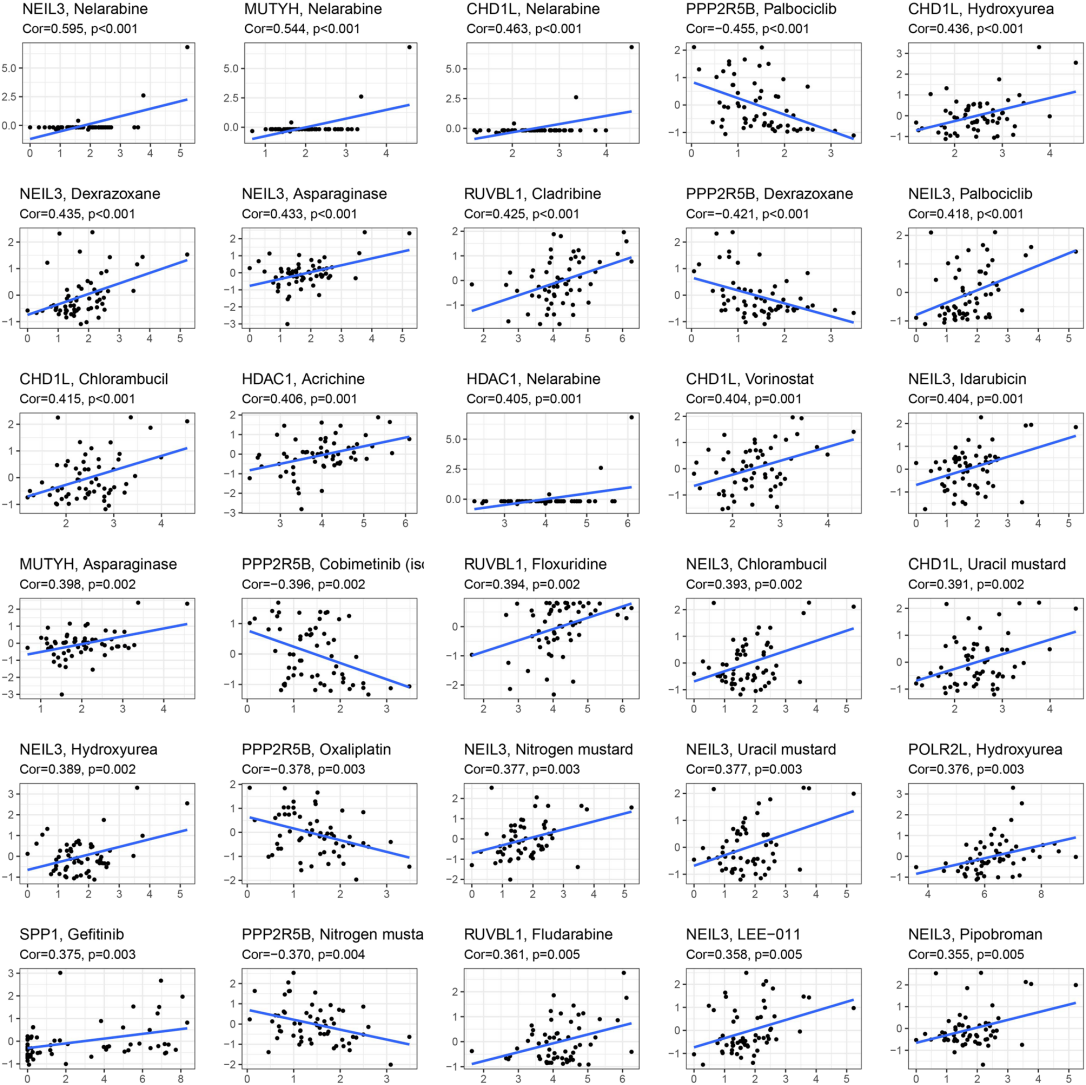
Scatter plot of the relationship between prognostic gene expression and drug sensitivity.

We investigated the association between prognostic gene expression levels and drug sensitivity in NCI-60 cell lines. Our analysis revealed that each prognostic gene was significantly associated with specific chemotherapeutic drug sensitivities (P < 0.01) (Fig. 8, Supplementary Table S6). For instance, higher levels of *CHD1L*, *HDAC1*, *MUTYH*, *NEIL3*, *POLR2L*, *RUVBL1*, and *SPP1* expression in cancer cells have been linked to higher levels of drug resistance to nelarabine, acridine, chlorambucil, dexrazoxane, cladribine, and other drugs. On the other hand, elevated *PPP2R5B* expression has been found to be associated with increased drug sensitivity of cancer cells to several chemotherapeutic agents, including oxaliplatin.

### Validation of prognostic gene expression in HCC and normal tissues

**Figure 9:**
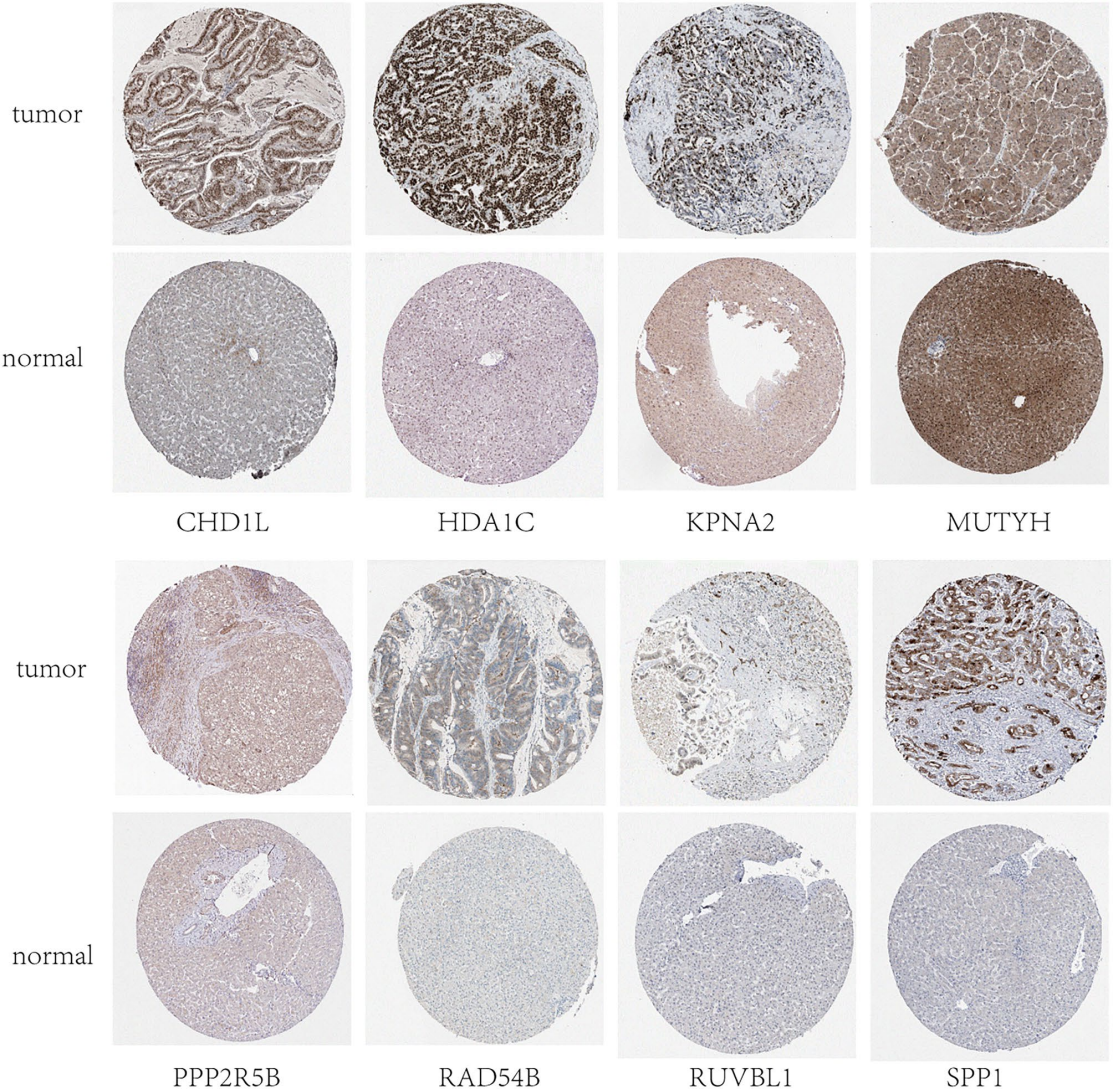
Protein expression of the eight genes in normal and HCC tissues based on Human Protein Atlas immunohistochemistry.

**Figure 10:**
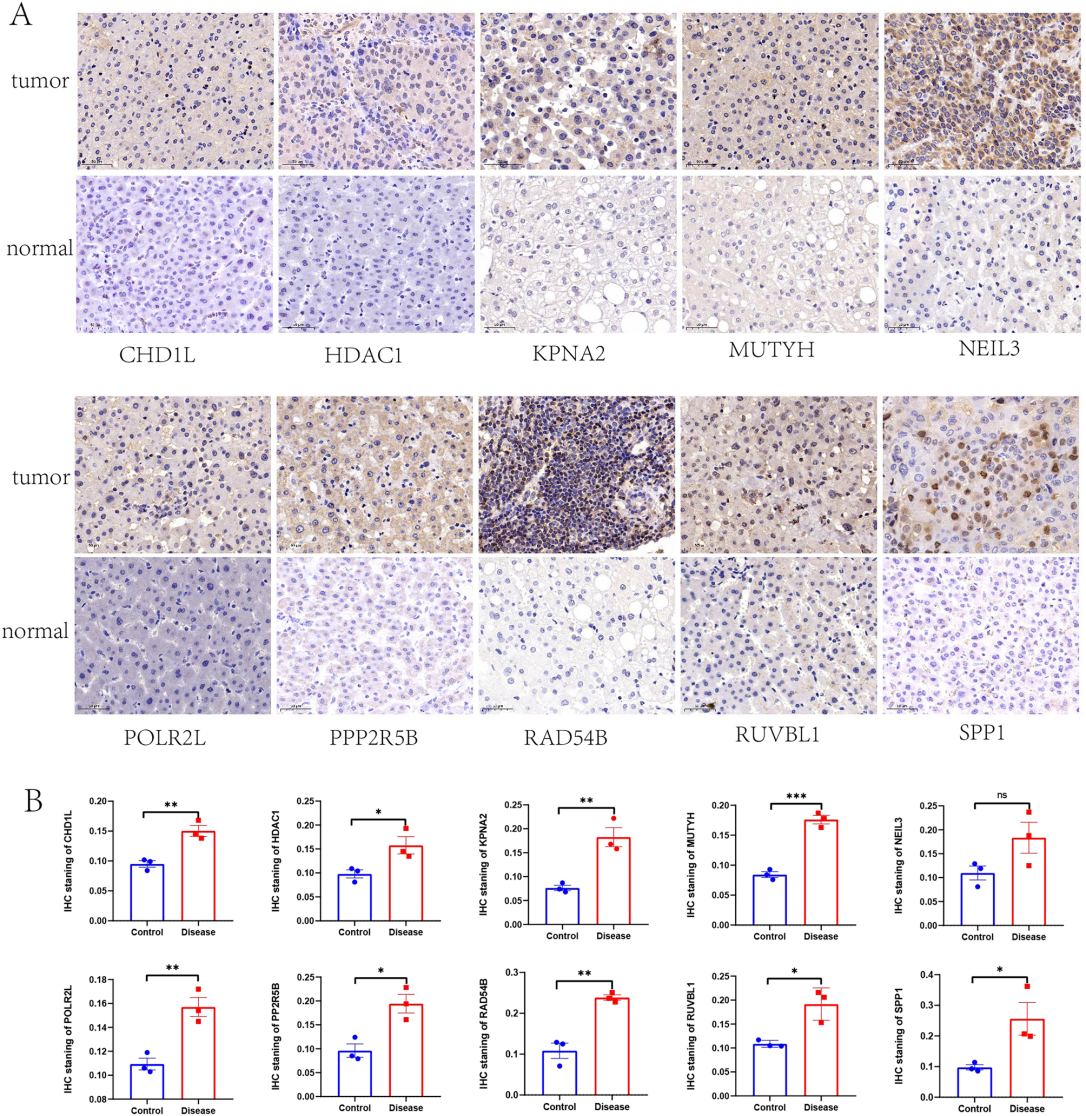
Protein expression levels of 10 prognostic genes in human HCC and normal liver tissues measured by IHC (*P < 0.05, **P < 0.01, ***P < 0.001). (*P < 0.05, **P < 0.01, ***P < 0.001, *NS* not significant).

**Figure 11:**
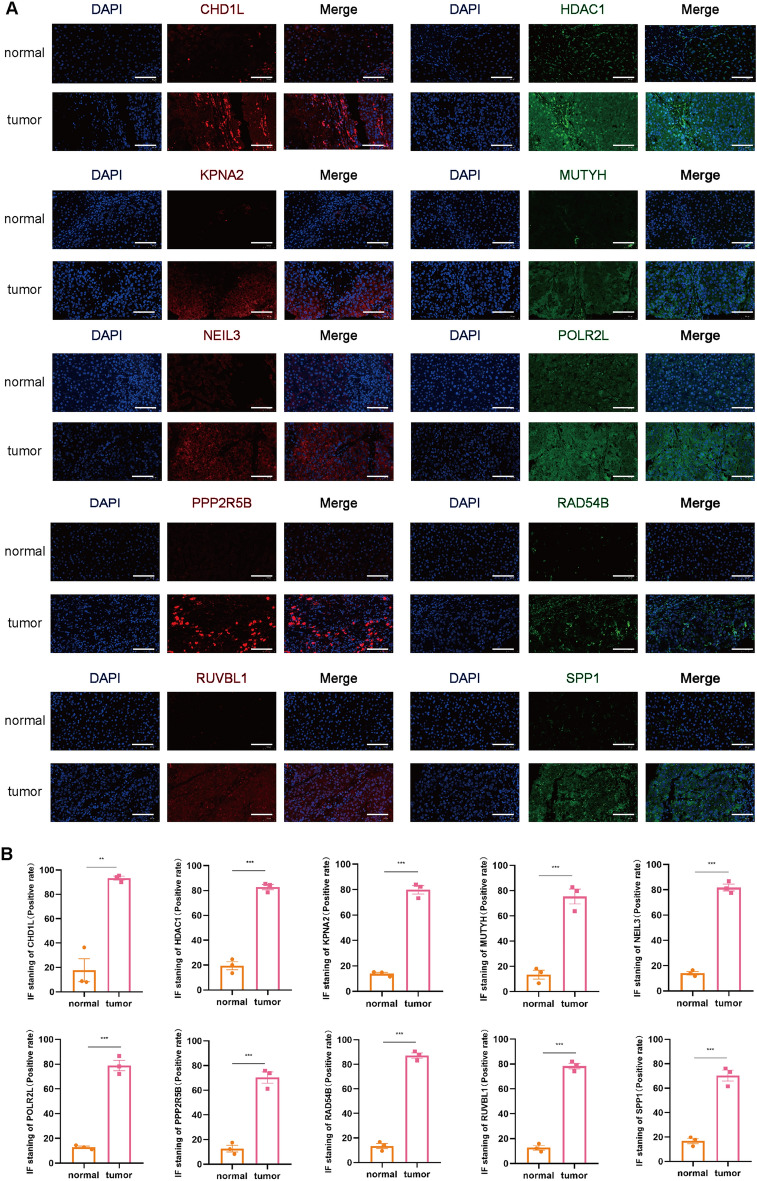
Protein expression levels of 10 prognostic genes in human HCC and normal liver tissues measured by IF (*P < 0.05, **P < 0.01, ***P < 0.001).

**Figure 12:**
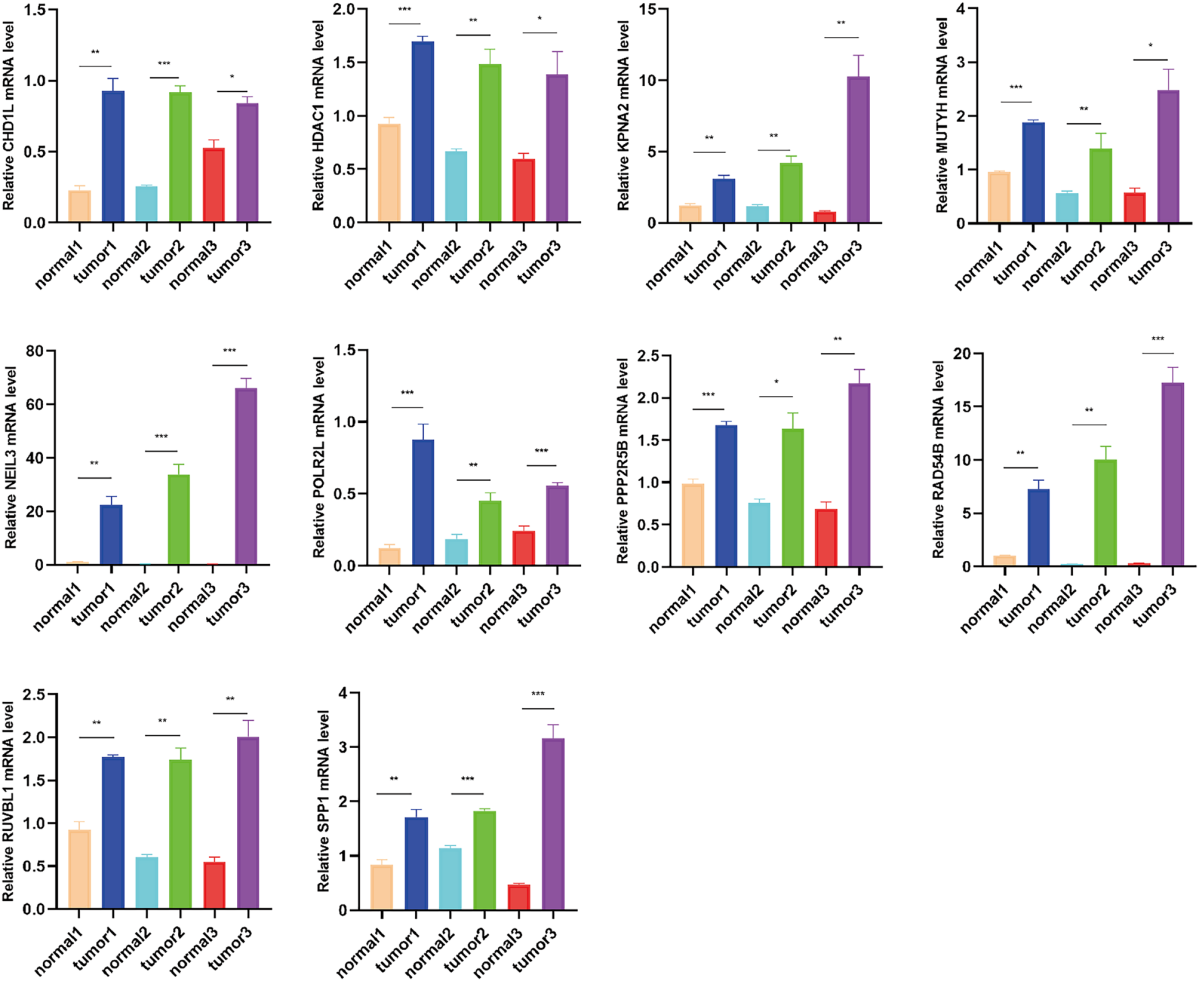
mRNA expression levels of 10 prognostic genes in mouse HCC and normal mouse liver tissues measured by qRT-PCR (*P < 0.05, **P < 0.01, ***P < 0.001).

According to the HPA database, the protein level of *MUTYH* was higher in normal tissues than in tumor tissues, and the protein expression levels of the seven genes (data for *NEIL3* and *POLR2L* were lacking and therefore not presented) were significantly higher in tumor samples than in normal samples (Fig. 9). Additionally, we further verified these 10 prognostic genes by IHC, IF, and qRT-PCR to confirm their expression in the protein and mRNA of hepatocellular carcinoma and normal liver tissue. The results of IHC showed that except for the expression of *NEIL3*, which was not statistically significant in normal liver tissues versus HCC tissues, the other 9 genes matched our bioinformatic predictions, and they were significantly overexpressed in HCC tissues compared to normal liver tissues (Fig. 10). As shown in Fig. 11, the expression of all 10 genes according to IF was significantly higher in HCC tissues than in normal liver tissues, which is consistent with our bioinformatics results. We hypothesized that the sample size might be too small, resulting in the expression of *NEIL3* not being statistically significant according to IHC. Therefore, to increase the credibility of the results, we constructed a mouse HCC model to further verify whether there is a difference in the expression of these 10 genes at the RNA level in mouse HCC tissues and normal mouse liver tissues by qRT-PCR. As shown in Fig. 12, at the mRNA level, these 10 genes were significantly expressed in HCC tissues and expressed at lower levels in normal liver tissues. These observations further confirm our conclusion that these genes are of high clinical significance as potential biomarkers for the diagnosis and prognosis of HCC.

## Discussion

With the establishment of next-generation sequencing technologies and the era of precision medicine approaches, a variety of treatments for HCC have been introduced. However, the small number of useful biomarkers often prevents the early diagnosis of HCC and prediction of the outcome of treatment. Several studies have demonstrated that novel serum biomarkers, such as a combination of circulating tumor cells, circulating nucleic acids and retinol and retinal panels, exhibit excellent prognostic accuracy in HCC^26–28^. Additionally, if DNA damage is not properly repaired and continues to accumulate, it might cause hepatocytes to change malignantly and ultimately result in HCC^29^. Therefore, DNA damage and repair (DDR) is a crucial molecular process for the emergence and progression of HCC, and more research on this process will establish the groundwork for an all-encompassing approach to treating HCC. However, the use of a gene profile associated with DDR as a prognostic indicator for HCC has not been documented. Our gene signature has higher accuracy in predicting prognosis, earlier studies have shown that gene signatures related to inflammatory, ferroptosis, immunity, m6A and energy metabolism in predicting 3-year OS in HCC only the immune-related gene signature had an AUC of > 0.70 in the experimental group, and the AUC of our DDRG signature was greater than 0.70 in both experimental and validation groups^28,30–33^. The DDRG signature created in our work exhibits greater benefits than the gene signature above, in addition to having high predictive performance for HCC prognosis. The algorithm is capable of categorizing immune checkpoint genes and tumor resistance genes into high- and low-expression groups. Additionally, several studies have demonstrated a significant correlation between risk scores and drug resistance to various chemotherapeutic agents.

From the TCGA cohort, we identified DEGs through a screening process. Further analysis using univariate Cox regression revealed 173 DEGs that were significantly associated with OS. By utilizing LASSO regression analysis, we developed a prognostic model that incorporated 10 DDRGs. We then evaluated this model using the ICGC cohort. Our analysis revealed that the high-risk group was significantly associated with a shorter OS, advanced TNM stage, and higher tumor grade. Moreover, independent prognostic analysis confirmed that the risk score was an independent predictor of OS, this is similar to previous findings that high repair groups tend to be accompanied by a worse prognosis^15^.

The prognostic model established in this study consisted of 10 DDRGs (*CHD1L*, *HDAC1*, *KPNA2*, *MUTYH*, *PPP2R5B*, *NEIL3*, *POLR2L*, *RAD54B*, *RUVBL1* and *SPP1*). A poor prognosis was nearly always linked to these genes. *CHD1L* is a newly discovered oncogene that has been linked to cancer via apoptosis inhibition, G1/S transition, and uncontrolled cell proliferation^34^. *HDAC1*-mediated inhibition of hepatocyte markers is a critical stage in the formation of hepatoblastoma, laying the groundwork for the development of treatments for aggressive hepatoblastoma by inhibiting *HDAC1* activity^35^. *KPNA2*, a nuclear transporter family member, has been recently shown to promote tumor growth and progression by involvement in cell differentiation, proliferation, apoptosis, immunological response, and viral infection^36^. *MUTYH* has been shown to be possibly associated with hepatocarcinogenesis in nonalcoholic steatohepatitis in a mouse model^37^. The role of *PPP2R5B* in liver cancer remains unclear. Some data suggest that deleting the gene causes paclitaxel sensitivity in cervical cancer and that this sensitivity shift is related to apoptosis^38^. Hui-huang Lai et al. demonstrated that *NEIL3* activated the BRAF/MEK/ERK/TWIST pathway-mediated EMT and therapeutic resistances, leading to HCC progression^39^. Single-cell RNA sequencing has revealed that *POLR2L* may contribute to the development of HCC through cell cycle-related pathways^40^. *RAD54B* has been investigated as a predictive marker for HCC patients, and it may play a significant role in the development of HCC through cancer cell DNA amplification^41^. *RUVBL1* is an AAA + ATPase whose expression is associated with a poor prognosis in HCC. On the other hand, the involvement of *RUVBL1* in the initial stages and growth of HCC is uncertain^42^. *SPP1*, which is a member of the SIBLING family, has been shown in some research to be overexpressed in numerous malignancies and can serve as a prognostic indicator^43^.

To gain insight into the interaction between the risk score and immune components, we examined the function of the risk score in the immune infiltration type. Our findings revealed that high risk scores were significantly associated with C1, whereas low risk scores were clearly associated with C3. These results suggest that C1 promotes tumorigenesis and progression, while C3 functions as a protective factor. This finding demonstrated that increased cytotoxicity might prevent tumor onset and growth^44^. Regarding the association between risk scores and clinical features, there was a significant correlation between high-risk scores and tumor grade 3–4 or tumor stage III–IV. These findings suggest that high risk scores are strongly associated with poor prognosis.

Despite the findings linking these genes to DNA damage repair, further research is necessary to determine their impact on the prognosis of HCC patients. Additionally, tumor-related signaling pathways, such as the cell cycle, phagosome, antigen binding, and organelle fission, were significantly enriched in the GO and KEGG analyses. The constant activation of these pathways has been associated with HCC, indicating potential novel treatment targets^45,46^. Furthermore, the high-risk group exhibited a higher number of macrophages and Treg cells. Previous research has revealed that an increase in tumor-associated macrophages and Treg cells is associated with poor prognosis in patients with HCC, likely due to their role in immune invasion^47–49^. Immunotherapy for cancer targeting immune checkpoints (e.g., anti-PD-L1 antibodies) has shown clinical activity in a variety of cancer types^50^. Immunotherapy based on immune checkpoint inhibitors has shown significant progress in the treatment of HCC. The expression of PD-1 and CTLA4 receptors inhibits the antitumor immunological response of T cells. As a result, the tumor can evade the body’s natural defenses and continue to grow. However, the use of immune checkpoint inhibitors has demonstrated significant therapeutic benefits in prolonging survival and improving quality of life for HCC patients^51^. Our study uncovered a strong correlation between the risk score and the expression of both PD-L1 and PD-1, whereby markedly higher scores were evident in the high-risk group than in the low-risk group. Our prognostic model therefore holds significant potential in facilitating accurate predictions of immune checkpoint expression levels and aiding in treatment decision-making. Furthermore, we found a distinct correlation between a high-risk score and a marked decrease in type II interferon (IFN) response activity. High levels of type II IFN response activity are central to tumor immune surveillance, as they stimulate antitumor immunity and support tumor elimination^44,52,53^. Additionally, our study identified increases in T-follicular helper (Tfh) cells, regulatory T (Treg) cells, T-helper 1 (Th1) cells, T-helper 2 (Th2) cells, and both T-cell costimulatory and T-cell coinhibitory activities within the high-risk group, which all point to a disruption in immune regulation within this cohort. Thus, we postulated that the diminished antitumor immunity was likely responsible for the poor prognosis found in the high-risk group.

Using NCI-60 cell line data, numerous prognostic genes were found to have increased expression associated with increased resistance to many FDA-approved chemotherapeutic agents. Of course, various prognostic genes were also correlated with increased sensitivity to a few drugs. In fact, immuno-oncology preclinical work has shown that combination immunotherapies including LAG-3 and CTLA4 blockade may have a synergistic effect on the anticancer immune response^54^. Based on these findings, several prognostic genes could serve as potential therapeutic targets to mitigate medication resistance or enhance susceptibility to adjuvant therapy.

In conclusion, the 10 DDRGs related with HCC prognosis that we analysed using the LASSO method offer fresh perspectives on how to treat HCC on an individual basis. We further confirmed by IHC, IF, and qRT-PCR that these 10 genes were differentially expressed in HCC tissues and normal tissues both at the protein level and mRNA level. All of them were highly expressed, and the experimentally verified expression trends were consistent with the findings of the bioinformatics analysis, which strengthened the validity of our findings. Furthermore, only a limited amount of research has been done on these 10 genes in HCC, and through our experiments, we have discovered that some of these genes are highly expressed in HCC for the first time. As a result, our prognostic model and experimental findings can offer fresh perspectives and a theoretical framework for future studies on HCC.

## Conclusion

In summary, our research identified a novel predictive signature comprising ten DDRGs. This signature was independently associated with OS and demonstrated utility in functional analysis, analysis of the tumor microenvironment, and prediction of medication sensitivity, providing novel insights for prognostic prediction in HCC. In addition, we verified by IHC, IF and qRT-PCR that the protein and mRNA expression of these ten DDRGs were all high in HCC and low in normal liver tissues. The particular underlying pathways connecting DDRGs and tumor immunity in HCC remain unknown, and our findings provide new directions for further investigation.

### Supplementary Information

Supplementary Figure S1.

Supplementary Figure S2.

Supplementary Figure S3.

Supplementary Figure S4.

Supplementary Figure S5.

Supplementary Figure S6.

Supplementary Table S1.

Supplementary Table S2.

Supplementary Table S3.

Supplementary Table S4.

Supplementary Table S5.

Supplementary Table S6.

Supplementary Legends.

## Data Availability

The data that support the fndings of this study are available from the corresponding author upon reasonable request.
