# Supplementary figures and images for: DNA damage repair-related gene signature for identifying the immune status and predicting the prognosis of hepatocellular carcinoma
Source: Sci Rep. 2023 Nov 3;13:18978. doi: 10.1038/s41598-023-45999-z (PMC10624694; doi:10.1038/s41598-023-45999-z)

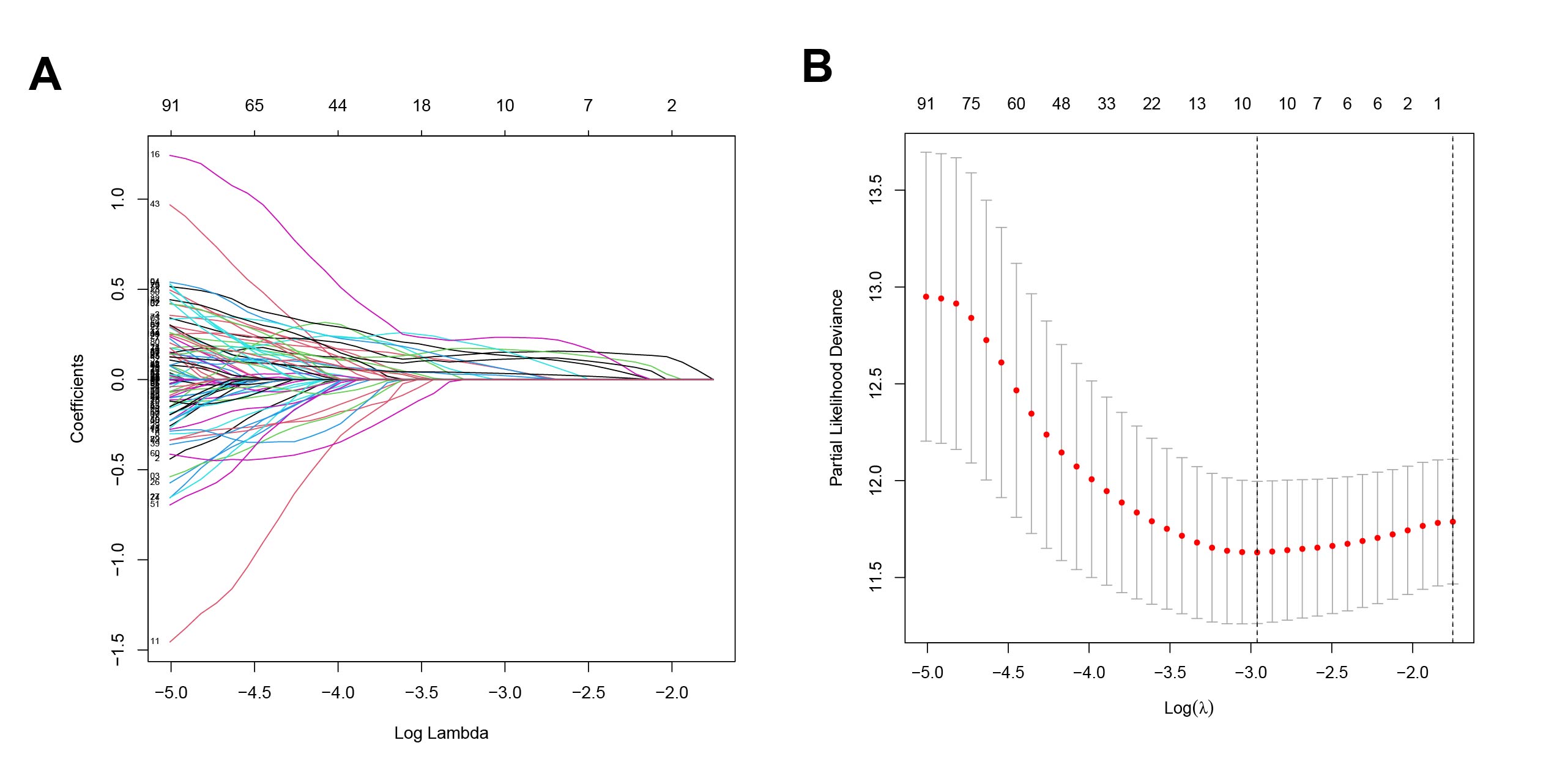

Supplement: Supplementary file 1 — Supplementary Figure S1. [file 41598_2023_45999_MOESM1_ESM.jpg]

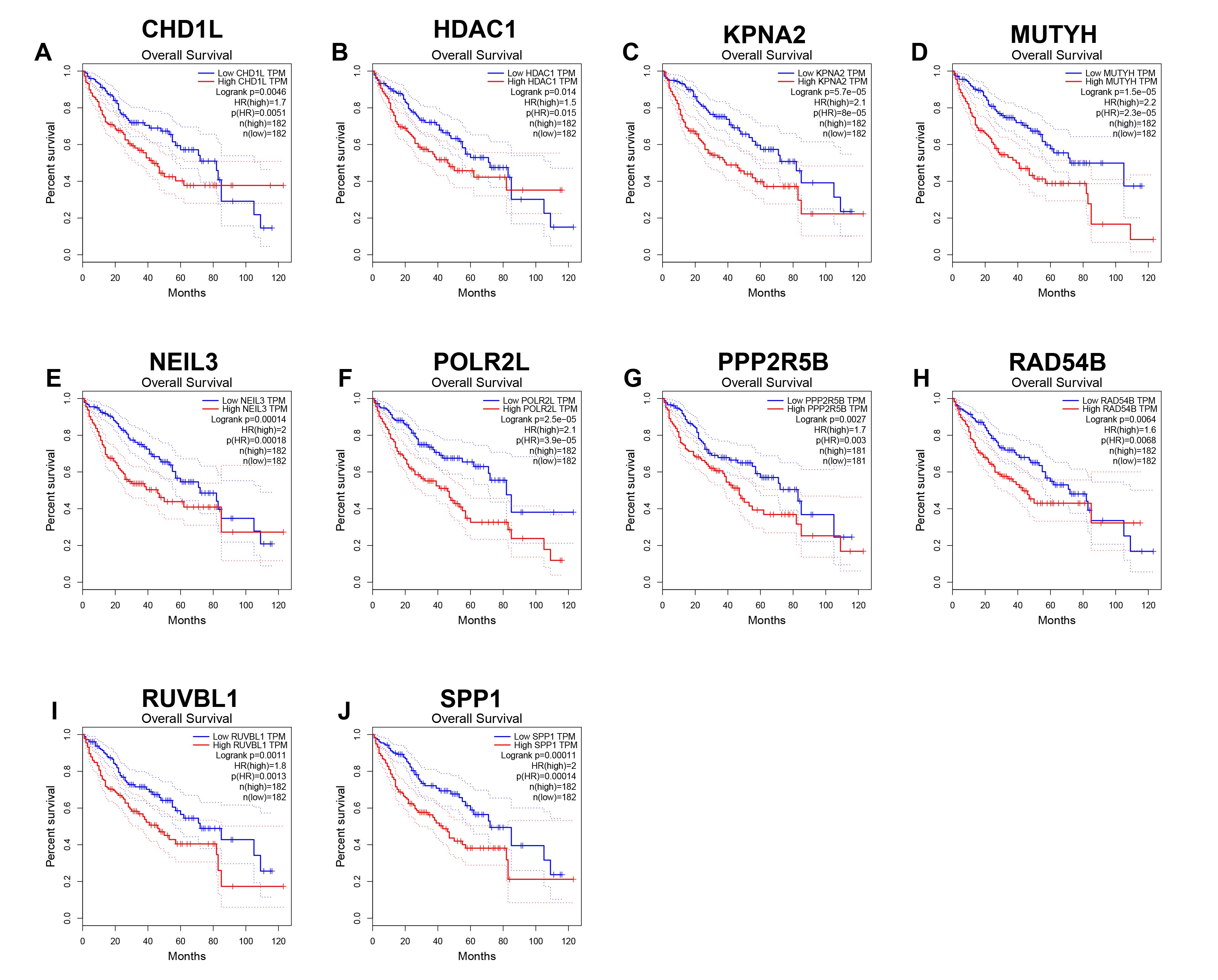

Supplement: Supplementary file 2 — Supplementary Figure S2. [file 41598_2023_45999_MOESM2_ESM.jpg]

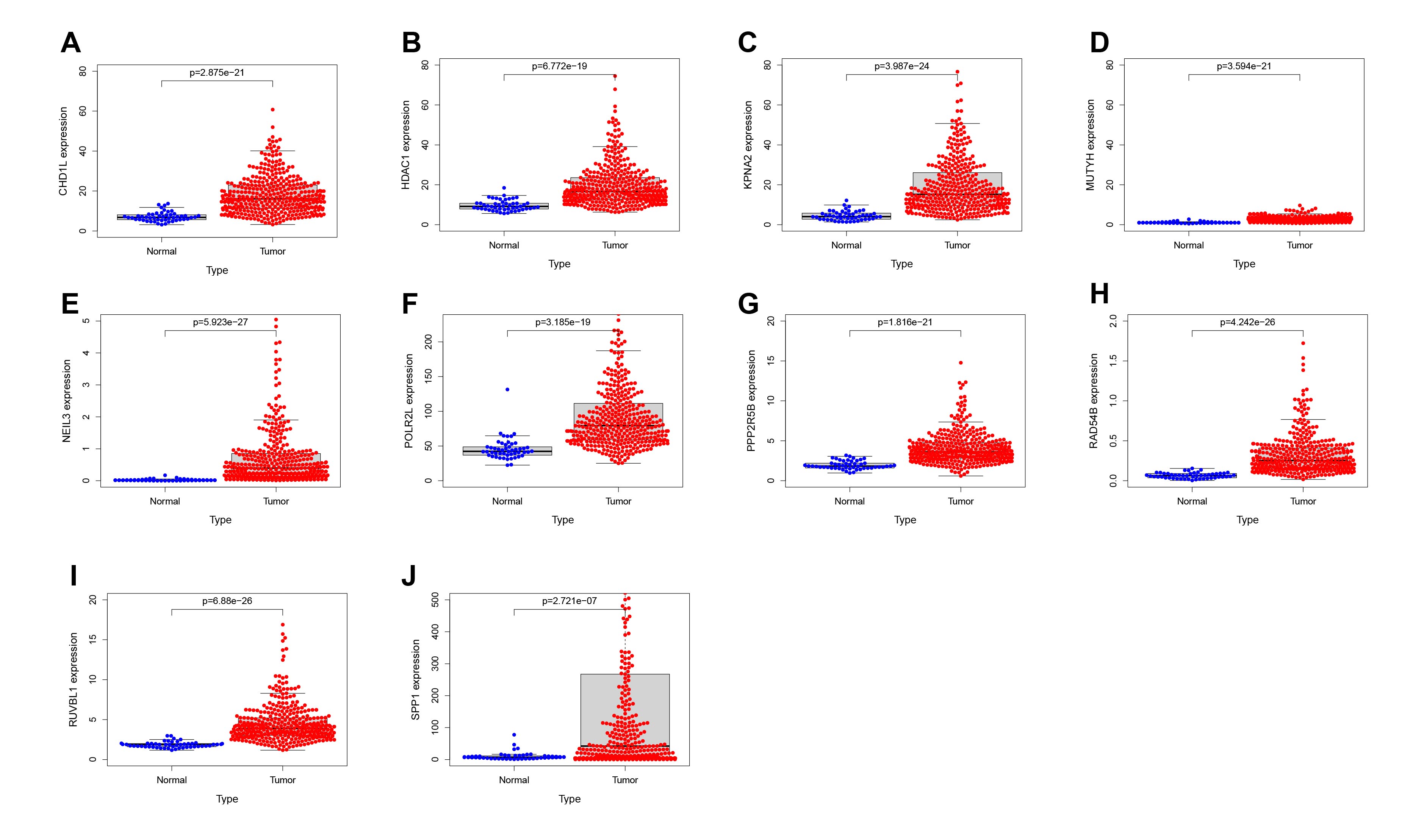

Supplement: Supplementary file 3 — Supplementary Figure S3. [file 41598_2023_45999_MOESM3_ESM.jpg]

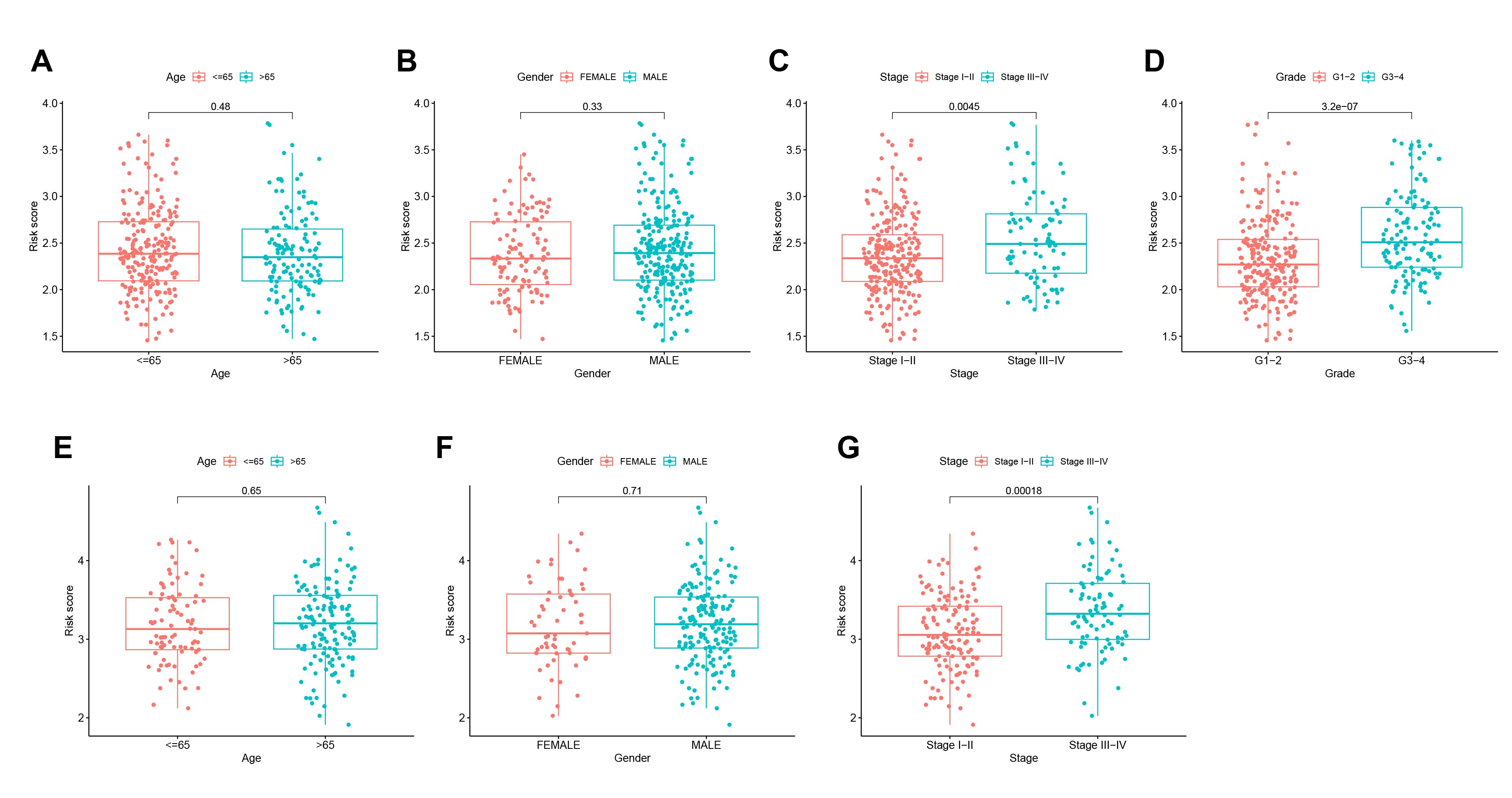

Supplement: Supplementary file 4 — Supplementary Figure S4. [file 41598_2023_45999_MOESM4_ESM.jpg]

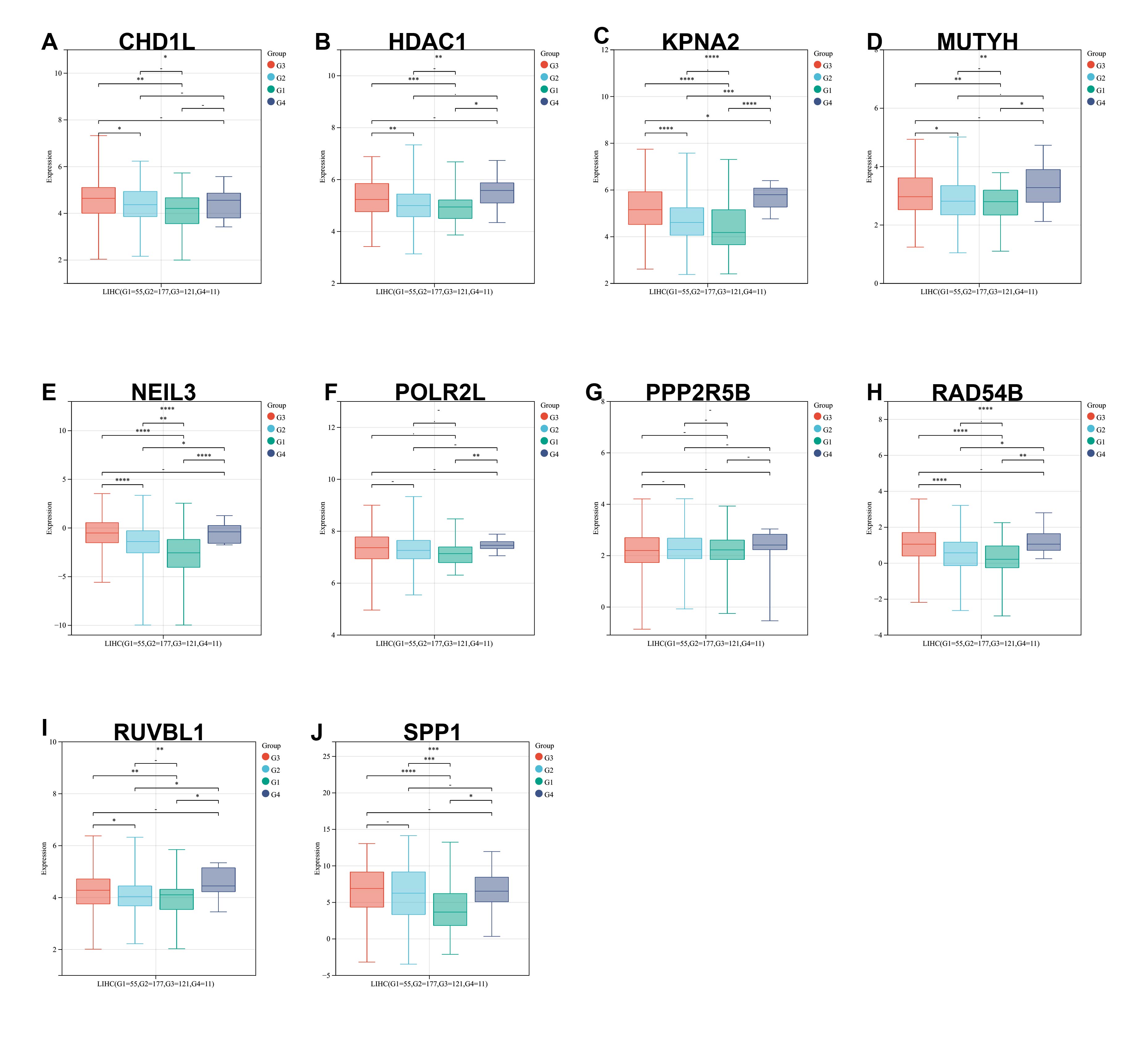

Supplement: Supplementary file 5 — Supplementary Figure S5. [file 41598_2023_45999_MOESM5_ESM.jpg]

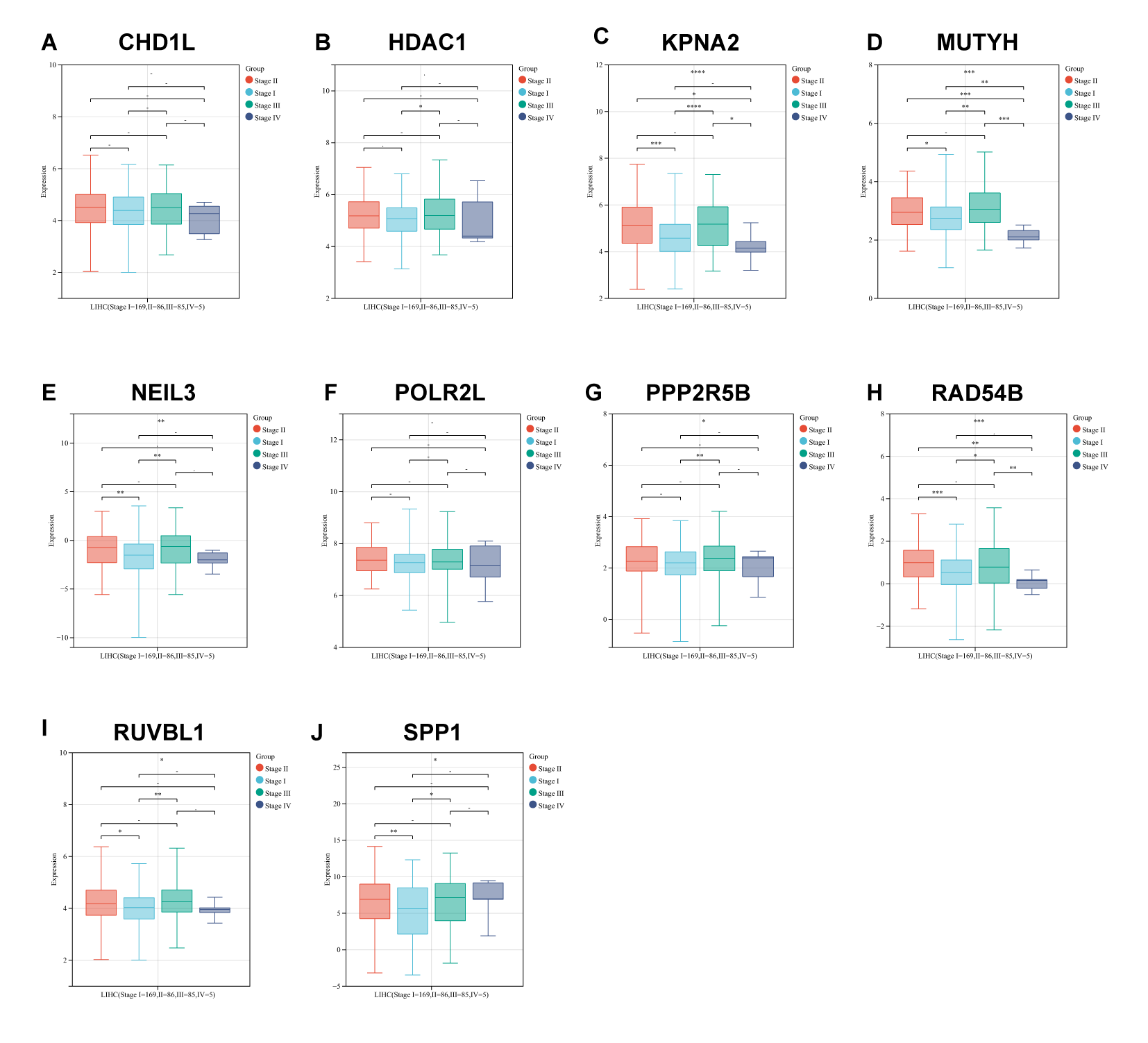

Supplement: Supplementary file 6 — Supplementary Figure S6. [file 41598_2023_45999_MOESM6_ESM.tif]
