# Supplementary material for: DNA damage repair-related gene signature for identifying the immune status and predicting the prognosis of hepatocellular carcinoma
Source: Sci Rep. 2023 Nov 3;13:18978. doi: 10.1038/s41598-023-45999-z (PMC10624694; doi:10.1038/s41598-023-45999-z)
Supplement: Supplementary file 7 — Supplementary Table S1. [file 41598_2023_45999_MOESM7_ESM.docx]

Supplementary Table S1: DNA damage repair genes (DDRGs)

| **Gene Names** |  |  |  |  |  |  |  |
| --- | --- | --- | --- | --- | --- | --- | --- |
| AATF  ABL1  ACTR5  AKT1  ALKBH1  ALKBH2  ALKBH3  AP5S1  AP5Z1  APEX1  APEX2  APITD1  APLF  APTX  ASCC3  ASF1A  ASTE1  ATF2  ATM  ATMIN  ATR  ATRIP  ATRX  ATXN3  AXIN2  BABAM1  BAP1  BARD1  BAX  BAZ1B  BCCIP  BLM  BRAP  BRCA1  BRCA2  BRCC3  BRE  BRIP1  BTG2  BUB1  BUB1B  C11orf30  C17orf70  C19orf40  CASP3  CCNA1  CCNA2  CCNB1  CCND1  CCNE1  CCNH  CCNO  CDC14B  CDC25A  CDC25B  CDC25C  CDC45  CDC6  CDH13  CDK1  CDK2  CDK4  CDK7  CDKN1A  CDKN1B  CDKN2A  CDKN2D  CEBPG  CEP164  CEP170  CETN2  CHAF1A  CHAF1B | CHD1L  CHD4  CHEK1  CHEK2  CHRNA4  CIB1  CINP  CLSPN  COPS5  CRB2  CREB1  CREBBP  CRY1  CRY2  CSNK1D  CSNK1E  CUL4A  CUL4B  CYP19A1  CYP1A1  DAPK1  DBF4  DCLRE1A  DCLRE1B  DCLRE1C  DDB1  DDB2  DDR1  DDX1  DEK  DHX9  DMAP1  DMC1  DNA2  DOT1L  DTL  DTX3L  DUSP3  DYRK2  E2F1  E2F2  E2F4  E2F6  EEPD1  EGFR  EME1  EME2  ENDOV  EP300  EPC2  ERBB2  ERCC1  ERCC2  ERCC3  ERCC4  ERCC5  ERCC6  ERCC6L2  ERCC8  ESCO1  ESCO2  ESR1  ETS1  EXO1  EXO5  EYA1  EYA2  EYA3  EYA4  FAM175A  FAN1  FANCA  FANCB | FANCC  FANCD2  FANCE  FANCF  FANCG  FANCI  FANCL  FANCM  FBXO18  FBXO6  FEN1  FGF10  FHIT  FIGN  FIGNL1  FOS  FOXM1  FTO  FZR1  GADD45A  GADD45G  GEN1  GPS1  GSTP1  GTF2H1  GTF2H2  GTF2H2C  GTF2H3  GTF2H4  GTF2H5  H2AFX  HDAC1  HDAC2  HELQ  HERC2  HIC1  HINFP  HIST3H2A  HMGB1  HMGB2  HUS1  HUS1B  HUWE1  IFI16  IGF1  IGHMBP2  IKBKG  INIP  INO80  INO80D  INO80E  INTS3  IRS1  JMY  JUN  KAT5  KDM2A  KIAA0101  KIAA0430  KIAA2022  KIF22  KIN  KPNA2  LIG1  LIG3  LIG4  MAD2L2  MBD4  MC1R  MCM9  MCPH1  MDC1  MDM2 | MDM4  MED17  MEIOB  MEN1  MGME1  MGMT  MLH1  MLH3  MMS19  MMS22L  MNAT1  MORF4L1  MORF4L2  MPG  MRE11A  MSH2  MSH3  MSH4  MSH5  MSH6  MTA1  MUM1  MUS81  MUTYH  MYC  NABP1  NABP2  NBN  NCOA6  NEIL1  NEIL2  NEIL3  NEK1  NEK11  NFKB1  NHEJ1  NINL  NME1  NONO  NSMCE1  NSMCE2  NTHL1  NUDT1  OGG1  OTUB1  PALB2  PAPD7  PARG  PARP1  PARP2  PARP3  PARP4  PARP9  PARPBP  PCNA  PLK1  PLK3  PMS1  PMS2  PNKP  POLA1  POLB  POLD1  POLD2  POLD3  POLD4  POLDIP3  POLE  POLE2  POLE3  POLE4  POLG  POLG2 | POLH  POLI  POLK  POLL  POLM  POLN  POLQ  POLR2A  POLR2B  POLR2C  POLR2D  POLR2E  POLR2F  POLR2G  POLR2H  POLR2I  POLR2J  POLR2K  POLR2L  PPM1D  PPP1CA  PPP2R2A  PPP2R5A  PPP2R5B  PPP2R5C  PPP2R5D  PPP2R5E  PPP4C  PPP4R2  PRKDC  PRMT6  PRPF19  PSMD3  PTTG1  RAD1  RAD17  RAD18  RAD21  RAD23A  RAD23B  RAD50  RAD51  RAD51AP1  RAD51B  RAD51C  RAD51D  RAD52  RAD54B  RAD54L  RAD9A  RAD9B  RASSF1  RB1  RBBP4  RBBP7  RBBP8  RBM14  RBX1  RDM1  REC8  RECQL  RECQL4  RECQL5  RELA  REV1  REV3L  RFC1  RFC2  RFC3  RFC4  RFC5  RFWD2  RFWD3 | RHNO1  RNASEH2A  RNF168  RNF169  RNF8  RPA1  RPA2  RPA3  RPA4  RPAIN  RPS27A  RPS27L  RPS3  RRM2B  RTEL1  RUVBL1  RUVBL2  SETD2  SETMAR  SETX  SFPQ  SFR1  SHFM1  SHPRH  SIRT1  SIRT6  SLC30A9  SLX1A  SLX4  SMAD2  SMAD3  SMAD4  SMAD7  SMARCA1  SMARCA2  SMARCA4  SMARCA5  SMARCAD1  SMARCB1  SMARCC2  SMARCD1  SMARCD2  SMC1A  SMC2  SMC3  SMC4  SMC5  SMC6  SMG1  SMUG1  SMURF2  SOD1  SP1  SPATA22  SPIDR  SPO11  SPP1  SPRTN  SSRP1  STAT1  STRA13  SUMO1  SUPT16H  SWI5  SWSAP1  SYCP1  TAOK1  TAOK2  TAOK3  TCEA1  TDG  TDP1  TDP2 | TELO2  TERF1  TERF2  TERF2IP  TEX12  TEX15  TICRR  TMEM161A  TNP1  TONSL  TOP1  TOP2A  TOP3A  TOPBP1  TP53  TP53BP1  TP73  TREX1  TREX2  TRIP12  TRIP13  TTC5  TWIST1  TYMS  UBA1  UBA52  UBB  UBC  UBE2A  UBE2B  UBE2D3  UBE2I  UBE2N  UBE2NL  UBE2T  UBE2U  UBE2V2  UBE4B  UHRF1  UIMC1  UNG  UPF1  USP1  USP28  USP3  USP47  USP7  UVRAG  UVSSA  VCP  WDR16  WDR33  WDR48  WEE1  WHSC1  WRN  WRNIP1  WWP1  WWP2  XAB2  XPA  XPC  XRCC1  XRCC2  XRCC3  XRCC4  XRCC5  XRCC6  XRCC6BP1  YY1  ZBTB32  ZFYVE26  ZNF350 | ZRANB3  ZSWIM7 |
