# Supplementary material for: DNA damage repair-related gene signature for identifying the immune status and predicting the prognosis of hepatocellular carcinoma
Source: Sci Rep. 2023 Nov 3;13:18978. doi: 10.1038/s41598-023-45999-z (PMC10624694; doi:10.1038/s41598-023-45999-z)
Supplement: Supplementary file 9 — Supplementary Table S3. [file 41598_2023_45999_MOESM9_ESM.docx]

**Table 1. Clinical characteristics of the HCC patients used in this study.**

|  | **TCGA-LIHC cohort** | **ICGC-LIRI-JP cohort** |
| --- | --- | --- |
| **No. of patients** | 365 | 231 |
| **Age (median, range)** | 57 (16-90) | 67 (31-89) |
| **Gender** |  |  |
| Female | 119 (32.6%) | 61 (26.4%) |
| Male | 246 (67.4%) | 170 (73.6%) |
| **Grade** |  |  |
| Grade 1 | 55 (15.1%) | NA |
| Grade 2 | 175 (47.9%) | NA |
| Grade 3 | 118 (32.3%) | NA |
| Grade 4 | 12 (3.3%) | NA |
| Unknown | 5 (1.4%) | NA |
| **Stage** |  |  |
| I | 170 (46.6%) | 36 (15.6%) |
| II | 84 (23.0%) | 105 (45.5%) |
| III | 83 (22.7%) | 71 (30.7%) |
| IV | 4 (1.1%) | 19 (8.2%) |
| Unknown | 24 (6.6%) | 0 (0%) |
| **Survival status** |  |  |
| Alive | 235 (64.4%) | 189 (81.8%) |
| Deceased | 130 (35.6%) | 42 (18.2%) |
