# Supplementary material for: DNA damage repair-related gene signature for identifying the immune status and predicting the prognosis of hepatocellular carcinoma
Source: Sci Rep. 2023 Nov 3;13:18978. doi: 10.1038/s41598-023-45999-z (PMC10624694; doi:10.1038/s41598-023-45999-z)
Supplement: Supplementary file 13 — Supplementary Legends. [file 41598_2023_45999_MOESM13_ESM.docx]

**Supplementary Material**

**Supplementary Figure S1**: A 10-gene signature was constructed in the TCGA cohort. **(A)** LASSO coefficient expression characteristics of candidate genes. **(B)** The penalty parameter (λ) in the LASSO model was selected through cross-validation.

**Supplementary Figure S2**: Survival analysis of prognostic genes based on online website GEPIA **(A-J)**. All adjusted P < 0.05.

**Supplementary Figure S3**: Expression of each prognostic gene between HCC tissues and adjacent nontumor tissues in the TCGA database.

**Supplementary Figure S4**: Risk scores for the different groups are divided by clinical features. **(A-D)** TCGA cohorts, **(E-G)** ICGC cohorts. **(A, E)** Age. **(B, F)** Sex. **(C, G)** Tumor stage. **(D)** Tumor grade.

**Supplementary Figure S5**: Expression of each prognostic gene in different groups

divided by tumor grade.

**Supplementary Figure S6**: Expression of each prognostic gene in different groups

divided by tumor stage.

**Supplementary Table S1**: DNA damage repair genes (DDRGs).

**Supplementary Table S2**: 263 FDA-approved drugs or drugs currently being studied in clinical trials.

**Supplementary Table S3**: Clinical characteristics of the HCC patients used in this study.

**Supplementary Table S4**: Kyoto Encyclopedia of Genes and Genomes (KEGG) pathway enrichment analysis of DEGs.

**Supplementary Table S5**: Gene Ontology (GO) functional analysis showing enrichment of DEGs.

**Supplementary Table S6**: The association between prognostic gene expression levels and drug sensitivity in NCI-60 cells.
